# Clinical Outcomes of Acute Myeloid Leukemia Patients Harboring the RUNX1 Mutation: Is It Still an Unfavorable Prognosis? A Cohort Study and Meta-Analysis

**DOI:** 10.3390/cancers14215239

**Published:** 2022-10-26

**Authors:** Tarinee Rungjirajittranon, Theerapat Siriwannangkul, Smith Kungwankiattichai, Nattawut Leelakanok, Wannaphorn Rotchanapanya, Pongthep Vittayawacharin, Benjamaporn Mekrakseree, Kamolchanok Kulchutisin, Weerapat Owattanapanich

**Affiliations:** 1 Division of Hematology, Department of Medicine, Faculty of Medicine Siriraj Hospital, Mahidol University, Bangkok 10700, Thailand; 2 Department of Clinical Pharmacy, Faculty of Pharmaceutical Sciences, Burapha University, Chonburi 20131, Thailand; 3 Department of Medicine, Chiangrai Prachanukroh Hospital, Chiangrai 57000, Thailand; 4 Division of Hematology, Department of Medicine, Rajavithi Hospital, Bangkok 10400, Thailand; 5 Faculty of Medicine Siriraj Hospital, Mahidol University, Bangkok 10700, Thailand

**Keywords:** acute myeloid leukemia, genetic, molecular, mutation, next-generation sequencing, *RUNX1*

## Abstract

**Simple Summary:**

Acute myeloid leukemia (AML) with mutated *RUNX1* (*RUNX1*^mut^) has an adverse prognosis based on the 2022 European LeukemiaNet risk stratification. However, the WHO classifications of 2022 removed *RUNX1* mutations from the unique entity because of various prognoses and treatment outcomes. Intriguingly, the overall survival (OS) and relapse-free survival (RFS) outcomes were similar in patients who had *de novo* AML with intermediate-risk cytogenetics with and without *RUNX1*^mut^. Our study endorsed an unfavorable prognosis of this entity.

**Abstract:**

Acute myeloid leukemia (AML) with mutated *RUNX1* (*RUNX1*^mut^) is considered to have an unfavorable prognosis. However, recent studies have reported comparable survival outcomes with wild-type *RUNX1* (*RUNX1*^wt^). To assess the clinical outcomes of AML with and without *RUNX1*^mut^, we performed a prospective cohort study and systematic review and meta-analysis. The study enrolled 135 patients (27 with *RUNX1*^mut^; 108 with *RUNX1*^w^^t^). There were no significant differences in the median OS and RFS of the *RUNX1*^mut^ and *RUNX1*^wt^ groups (9.1 vs. 12.2 months; *p* = 0.268 and 7.8 vs. 14.6 months; *p* = 0.481, respectively). A subgroup analysis of *de novo* AML patients with intermediate-risk cytogenetics showed similar outcomes. Our meta-analysis pooled data from 23 studies and our study. The complete remission rate was significantly lower in the *RUNX1*^mut^ group (pooled odds ratio: 0.42). The OS, RFS, and event-free survival rates also favored the *RUNX1*^wt^ group (pooled risk ratios: 1.36, 1.37, and 1.37, respectively). A subgroup analysis of *de novo* AML patients with intermediate-risk cytogenetics demonstrated nearly identical OS and RFS outcomes. This study confirms that patients with AML and *RUNX1*^mut^ had poor prognoses. Nonetheless, in *de novo* AML with intermediate-risk cytogenetics, the survival outcomes of both groups were comparable.

## 1. Introduction

Acute myeloid leukemia (AML) is a hematologic malignancy that results from impaired proliferation and differentiation of hematopoietic stem cells, leading to an accumulation of abnormal blast cells in bone marrow [1]. AML’s high heterogeneity is reflected in different disease prognoses, which are influenced by host- and disease-related factors [1,2,3]. For instance, old age, multiple comorbidities, and secondary AML are unfavorable predictive factors and the main obstacles for candidates of intensive therapy [1]. With regard to disease-related factors, the most essential one is molecular abnormalities [4]. The 2022 European LeukemiaNet (ELN) recommendations emphasize that cytogenetic and molecular studies are mandatory for AML diagnosis [5]. Molecular testing helps define risk stratification and identify targeted therapy [5].

The *RUNX1* gene, located on chromosome 21 (21q22), regulates human hematopoiesis of all lineages [6]. The gene has a variety of biological functions, including cell differentiation, proliferation, cell cycle, DNA repair, apoptosis, ribosomal biogenesis, and metabolism [7]. *RUNX1* is a recommended gene mutation that should be screened for in AML patients at diagnosis [5]. In 2016, the World Health Organization (WHO) classifications introduced “AML with mutated *RUNX1* (*RUNX1*^mut^)” as a provisional entity to AML with a recurrent genetic abnormalities subtype because *RUNX1*^mut^ patients had distinct clinical and genetic markers [8,9,10]. More recently, the 2022 International Consensus Classification stated that patients with *RUNX1* mutations fit in the entity of AML with myelodysplasia-related gene mutations and remain in the adverse risk group [5]. However, the WHO classifications of 2022 eliminated *RUNX1* mutations from the unique entity, instead defining a standalone AML type due to various clinical and molecular features [11]. A previous meta-analysis demonstrated that AML patients with *RUNX1*^mut^ had increased risks of developing discouraging survival outcomes [12]. However, several recent publications have shown different survival outcomes [13,14,15]. For instance, Quesada et al. demonstrated that the overall survival (OS) and event-free survival (EFS) rates of AML patients with and without *RUNX1*^mut^ were comparable [14]. Similarly, Venugopal et al. showed that both groups had similar OS rates, even among young and old patients [15].

We aimed to determine the clinical outcomes for a Southeast Asian population, for which there are presently limited data. Due to the conflicting results from previous studies, we performed a prospective study to illustrate a survival analysis of Thai AML patients with *RUNX1*^mut^ and *RUNX1* wild-type (*RUNX1*^wt^). Furthermore, we performed a systematic review and meta-analysis to better understand the characteristics and prognosis of overall AML patients with *RUNX1*^mut^ in real-world situations.

## 2. Materials and Methods

This was a prospective, single-center, observational study of newly diagnosed AML patients. We also conducted a systematic review and meta-analysis that integrated our findings with all published studies’ findings. This approach allowed us to comprehensively compare the clinical outcomes of patients with *RUNX1*^mut^ and those with *RUNX1*^wt^.

### 2.1. Prospective Cohort Study

The study was conducted on newly diagnosed AML patients with and without *RUNX1*^mut^ between January 2019 and April 2022. The patients attended Siriraj Hospital, Mahidol University, Thailand, an academic university hospital and the country’s largest acute leukemia referral center. Patients were enrolled if they were over 18 years old and had a diagnosis of AML requiring treatment and follow-up. We excluded patients diagnosed with acute promyelocytic leukemia or those who had not undergone molecular testing before treatment.

Our center’s next-generation sequencing technique examined entire coding regions of 25 genes recurrently mutated in myeloid neoplasms. These genes were as follows: *ASXL1*, *CALR*, *CBL*, *CEBPA*, *CSF3R*, *DNMT3A*, *EZH2*, *FLT3*, *IDH1*, *IDH2*, *JAK2*, *KIT*, *KRAS*, *MPL*, *NPM1*, *NRAS*, *RUNX1*, *SETBP1*, *SF3B1*, *SH2B3*, *SRSF2*, *TET2*, *TP53*, *U2AF1*, and *ZRSR2*. The initial process was DNA extraction using a QIAamp DNA Mini Kit (Qiagen, Hilden, Germany). The concentration of extracted genomic DNA (gDNA) was measured using a Nanodrop System (Thermo Fisher Scientific, Waltham, MA, USA) and a Qubit dsDNA HS assay (Qubit 3.0 Fluorimeter; Life Technologies, Carlsbad, CA, USA). The GeneRead QIAact Custom Panel (Qiagen, Hilden, Germany) was used in the QIAGEN GeneReader Next-Generation Sequencing System. The heterozygous variant allele frequency (VAF) detection cutoff was at least 3%. Each variant was manually analyzed and curated by the UCSC Genome Browser, the COSMIC database, and dbSNP [16,17,18]. DNA-based assays were also investigated in *NPM1* and *FLT3*-ITD, as reported by Stone et al. [19].

The Siriraj Institutional Review Board approved this research, which followed the Declaration of Helsinki guidelines and all subsequent amendments. All patients gave informed consent for biobanking and the analyses. The study was registered at the Thai Clinical Trial Registry (TCTR20220921005).

The primary outcome of this study was the OS rate. The secondary outcomes were the complete remission (CR) and relapse-free survival (RFS) rates, and significant factors associated with OS and RFS.

#### Statistical Analysis for the Cohort Study

All data analyses were designed a priori and performed using IBM SPSS Statistics for Windows, version 20.0 (Armonk, NY, USA: IBM Corp.). The sample size calculation was based on the OS rate from our previous pilot study. Details of the calculation are demonstrated in Supplementary Data S1. Demographic and baseline characteristics were summarized descriptively using 2 categories (*RUNX1*^mut^ and *RUNX1*^wt^ AML). Continuous variables are presented as medians and interquartile ranges or means ± standard deviations, depending on the data type. The Mann–Whitney U test was employed to compare continuous data. Categorical data are presented as numbers and percentages and compared using Fisher’s exact or Chi-squared tests. A log-rank test was used to compare the factors correlated with OS and RFS, and the results are presented as a Kaplan–Meier survival curve. Cox proportional hazards analysis was used to compare the predictors of survival outcomes in the univariate and multivariate analyses. The independent variables with significance in the univariate analysis were entered into the multivariate model. The results are expressed as hazard ratios (HRs) and 95% confidence intervals (CIs). Statistical significance was determined as a probability (*p*) value of <0.05.

### 2.2. Systematic Review and Meta-Analysis

#### 2.2.1. Data Sources and Searches

Three investigators (T.R., W.O., and T.S.) independently searched for published articles indexed in the MEDLINE, Embase, and Cochrane Library databases from inception to April 2022. The search strategy used the terms “acute myeloid leukemia” and “molecular”. Supplementary Data S2 details the strategy for each database. The study was performed following the PRISMA (Preferred Reporting Items for Systematic Reviews and Meta-analysis) statement (Supplementary Data S3) [20].

#### 2.2.2. Selection Criteria and Data Extraction

To qualify for the meta-analysis, studies had to be either randomized controlled studies or cohort studies (prospective or retrospective) and have 2 groups of patients. The first group was *RUNX1*^mut^ patients, and the other group was patients with *RUNX1*^wt^. Selected studies needed to report at least 1 of these outcomes: CR, OS, RFS, or EFS rates. Subgroup analyses included patients with *de novo* AML and those with intermediate-risk stratification, according to the 2017 ELN recommendations [21]. Three investigators (T.R., W.O., and T.S.) independently selected relevant articles and performed data extractions. Disagreements or questions regarding the eligibility of individual articles were discussed between the 3 investigators until a consensus was reached. Two investigators (T.R. and W.O.) subsequently examined the baseline characteristic data and the outcomes of all included studies, with the extracted data cross-checked to avoid inaccuracies.

#### 2.2.3. Quality Assessment

Quality assessment and risk of bias were assessed using Risk of Bias in Nonrandomized Studies of Interventions (ROBINS-I) [22].

#### 2.2.4. Statistical Analysis for the Meta-Analysis

Review Manager 5.4 software from the Cochrane Collaboration (London, UK) was used to analyze the data. The effect was estimated and combined with 95% CIs using the Mantel–Haenszel method [23]. Cochran’s Q statistic was calculated, and the statistical heterogeneity among the studies was estimated using the I^2^ statistic. The 4 levels of heterogeneity were based on the value of I^2^ as follows: (1) insignificant heterogeneity (I^2^ of 0–25%); (2) low heterogeneity (I^2^ of 26–50%); (3) moderate heterogeneity (I^2^ of 51–75%); and (4) high heterogeneity (I^2^ of 76–100%) [24]. A random-effects model was applied based on the assumption that there was heterogeneity in each study due to individual patient characteristics, treatment differences, and disease risk stratification [24]. *p* values less than 0.05 were considered statistically significant. Funnel plots and Egger regression were used to detect publication biases [25]. The study protocol was registered with the International Prospective Register of Systematic Review (PROSPERO) network (CRD42022327857).

#### 2.2.5. Terminology

Our patients’ diagnoses and risk stratification were based on cytogenetic and molecular findings according to the 2017 ELN classifications [21]. Secondary AML in this cohort consisted of cases of AML that had an antecedent hematological disorder, prior chemotherapy, or radiation therapy [26]. CR was defined as bone marrow blasts below 5%, the absence of extramedullary blasts, blasts with extramedullary disease with an absolute neutrophil count exceeding 1000/μL, and a platelet count of more than 100,000/μL [21]. The duration between diagnosis and either death or the last follow-up was defined as OS. The duration from the CR date to relapse or death from any cause gave the RFS. The EFS duration was measured from the date of diagnosis to treatment failure, disease relapse, or death from any cause [27].

## 3. Results

### 3.1. Prospective Cohort Study

The study enrolled 135 patients divided into two groups based on the presence of *RUNX1* mutation. Among them, 27 patients (20%) were *RUNX1*^mut^ patients, while 108 (80%) were *RUNX1*^wt^ patients. The cohort’s median age was 55 years (interquartile range (IQR): 40–64). However, the patients in the *RUNX1*^mut^ group were significantly older than those in the *RUNX1*^wt^ group, with median ages of 62 years (IQR: 55–75) and 53 years (IQR: 40–61), respectively (*p* = 0.008). The proportions of men and women in both groups were approximately equal. Most patients had an intermediate cytogenetic risk (73.3%) according to the 2017 ELN classifications. The most common subtype of AML was *de novo* AML (81.5%). However, the *RUNX1*^mut^ group had a higher proportion of secondary AML patients than *de novo* AML (40.7% and 13%). The baseline mutational status of patients showed the presence of *FLT3*-ITD (22.2%), *DNMT3A* (22.2%), *NPM1* (17.3%), *TP53* (9.6%), *IDH2* (8.1%), biallelic *CEBPA* (5.2%), *EZH2* (5.2%), *IDH1* (4.4%), and *ASXL1* (4.4%). Among the cases, 92 were treated with intensive chemotherapy regimens, consisting of 3 + 7 or 2 + 5 regimens, while 37 were treated with low-intensity therapy (hypomethylating agents, low-dose cytarabine, hydroxyurea, or palliative treatment). Only 16 patients (11.9%) from the entire cohort underwent allogeneic stem cell transplantation due to limited access to available matched donors in Thailand. None of the patients in our cohort received targeted therapies (FLT3, IDH1, IDH2, and BCL2 inhibitors). Table 1 summarizes the patients’ baseline characteristics, laboratory investigations, and treatments. The molecular mutation profiles of the *RUNX1*^mut^ and *RUNX1*^wt^ groups are compared in Supplementary Data S4.

#### 3.1.1. Treatment Responses and Clinical Outcomes

**Figure 1:**
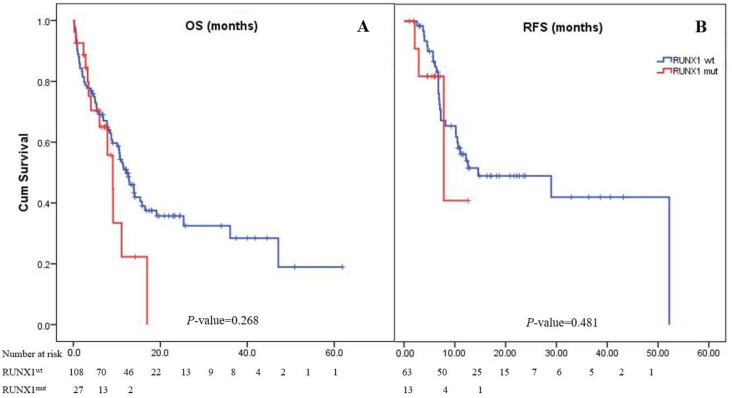
Kaplan–Meier curves of overall survival (**A**) and relapse-free survival (**B**). Panel A shows Kaplan–Meier curves for overall survival corresponding to the presence or absence of the *RUNX1* mutation. The median overall survival of AML patients with *RUNX1*^mut^ was 9.1 months, while it was 12.9 months in patients with *RUNX1*^wt^ (*p* = 0.268). Panel B shows Kaplan–Meier curves for relapse-free survival as assessed by the investigator. The median relapse-free survival was 7.8 months in the *RUNX1*^mut^ group and 14.6 months in the *RUNX1*^wt^ group (*p* = 0.481).

Most patients (82.6%) achieved CR after induction therapy. The CR rate in the *RUNX1*^mut^ group was 68.4%, whereas the *RUNX1*^wt^ group had a rate of 86.3% (pooled odds ratio (OR): 0.34; 95% CI: 0.11–1.11; *p* = 0.075). However, 52 (38.5%) patients experienced disease relapse (Table 1). The median follow-up period of the 135 patients in the study cohort was 2 years. The median OS of the patients in the cohort was 11.4 months (95% CI: 9.1–14.1), while the 1- and 2-year OS rates were 48% and 33%, respectively. The OS of patients with *RUNX1*^mut^ (9.1 months [4.0–11.1]) was comparable to that of the *RUNX1*^wt^ group (12.9 months [9.0–15.8]; *p* = 0.268; Figure 1). The overall RFS in this cohort was 14.6 months (95% CI: 0–31.7). The *RUNX1*^mut^ AML patients had a slightly shorter RFS than the *RUNX1*^wt^ patients (7.8 months [0.9–14.8] vs. 14.6 months [0–31.8]; *p* = 0.481; Figure 1).

#### 3.1.2. Factors Associated with Survival Outcomes

Comparing the factors associated with the OS of the patients from the multivariate analysis, *FLT3*-ITD, *DNMT3A,* *EZH2, IDH1*, and *TP53* mutations were associated with poor prognoses, with HRs of 2.74, 2.19, 3.93, 3.72, and 3.15, respectively. Patients older than 60 years had poorer prognoses than those under 60 years (HR: 2.80; 95% CI: 1.44–5.45; *p* = 0.002). *RUNX1* mutation was not associated with OS in our patients (HR: 1.42; 95% CI: 0.77–2.62; *p* = 0.268; Table 2).

Patients aged more than 60 years and the presence of poor-risk cytogenetics, the *FLT3*-ITD mutation, or the *SRSF2* mutation were significantly associated with poorer outcomes in the RFS analysis. However, *RUNX1* mutation had no impact on RFS (HR: 1.37; 95% CI: 0.41–4.62; *p* = 0.604; Table 2). Some factors influencing RFS outcomes might not have reached statistical significance due to the limited number of patients.

### 3.2. Systematic Review and Meta-Analysis

#### 3.2.1. Study Identification, Selection, and Characteristics

We searched for potentially relevant articles published up to April 2022 in the Embase (21 058 articles), Medline (23 470 articles), and Cochrane databases (730 articles) and screened them for retrieval. Of these articles, 14 831 were excluded due to duplication, leaving 30 427 articles. Three investigators (T.R., W.O., and T.S.) performed title and abstract reviews of these papers. Articles were excluded if they met at least one of these three criteria: (1) their population differed from that evaluated in our study; (2) they did not fulfill the inclusion criteria; and (3) they did not report our outcomes of interest. This process eliminated 30 202 articles. The 225 remaining articles underwent full-length article reviews. In total, 24 studies were eventually included in our meta-analysis: 12 prospective studies [9,13,14,28,29,30,31,32,33,34,35,36], 11 retrospective studies [37,38,39,40,41,42,43,44,45,46,47]), and our prospective cohort study. Supplementary Data S5 illustrates the literature review and article selection process.

Funnel plots of the CR, OS, RFS, and EFS outcomes of the AML patients with *RUNX1*^mut^ and *RUNX1*^wt^ were relatively symmetrical and showed no publication bias (Supplementary Data S6). We did not conduct any other funnel plot symmetrical tests since such tests are not recommended when the standard errors of the intervention effect estimates are approximate [48]. For other biases, ROBINS-I was used for the assessments (Supplementary Data S7).

#### 3.2.2. Baseline Patient Characteristics

A total of 8022 patients were included: 1093 *RUNX1*^mut^ AML patients and 6929 *RUNX1*^wt^ AML patients. The age of the participants varied markedly in both groups (from 11 to 92 years). Most patients (92.2%) had *de novo* AML, while 7.8% had secondary AML. According to the 2017 ELN risk classifications, the participants comprised 646 patients with favorable risk, 3861 patients with intermediate risk, and 1404 patients with poor risk. However, there were 2111 patients whose prognostic details were unavailable. Three common molecular mutations were the *NPM1* mutation, *FLT3*-ITD mutation, and *DNMT3A* mutation. Most patients received standard induction chemotherapy, including the 3 + 7 regimen and the high-dose cytarabine regimen. The characteristics of the studies and patients are summarized in Table 3.

#### 3.2.3. Treatment Response and Clinical Outcomes

**Figure 2:**
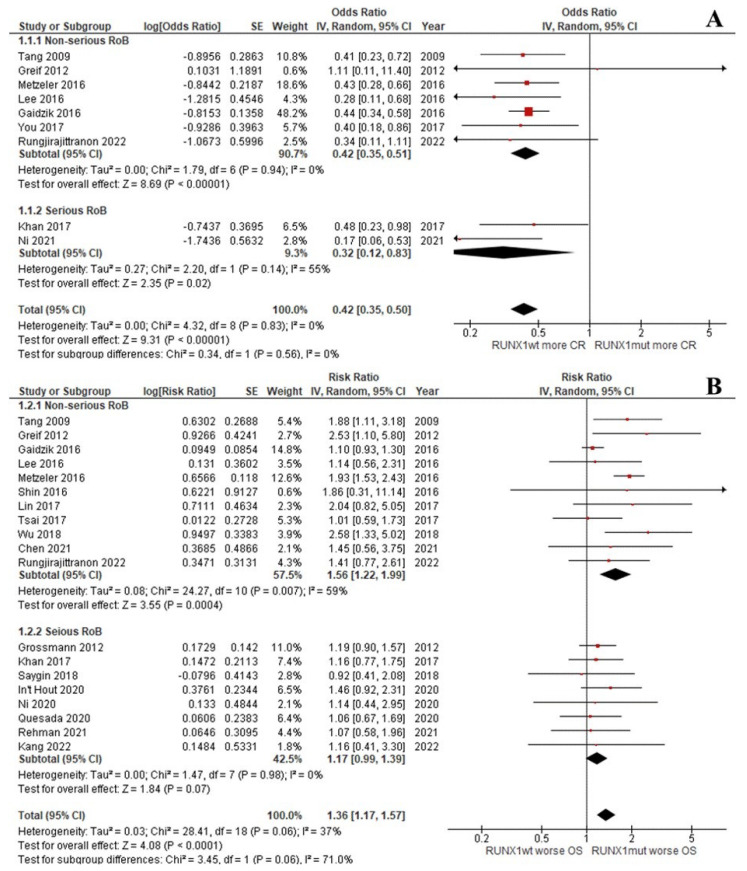
Forest plot of the clinical outcomes of the *RUNX1*^mut^ and *RUNX1*^wt^ AML patients: (**A**) CR rate; (**B**) OS.

**Figure 3:**
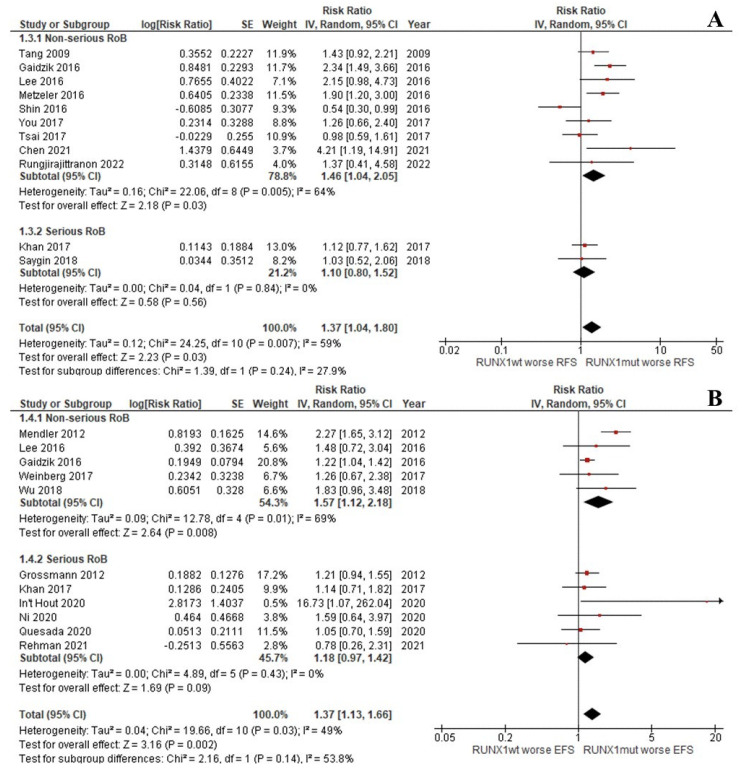
Forest plot of the clinical outcomes of the *RUNX1*^mut^ and *RUNX1*^wt^ AML patients: (**A**) RFS; (**B**) EFS.

The CR rate of the patients with *RUNX1*^mut^ was significantly lower than that of the *RUNX1*^wt^ patients (OR: 0.42; 95% CI: 0.35–0.50; I^2^ = 0%; *p <* 0.00001; Figure 2A) [9,13,29,31,33,37,39,45]. Likewise, the OS, RFS, and EFS rates of the *RUNX1*^mut^ group were significantly inferior to the corresponding values of *RUNX1*^wt^ patients. For OS, the pooled risk ratio (RR) was 1.36 (95% CI: 1.17–1.57; I^2^ = 37%; *p <* 0.0001; Figure 2B) [9,13,14,29,31,32,34,35,36,37,38,39,40,42,43,44]. For RFS, the RR was 1.37 (95% CI: 1.04–1.80; I^2^ = 59%; *p* = 0.03; Figure 3A) [9,13,29,33,34,37,38,39,42,44]. For EFS, the RR was 1.37 (95% CI: 1.13–1.66; I^2^ = 49%; *p* = 0.002; Figure 3B) [9,13,30,32,35,36,37,41,43,46].

#### 3.2.4. Subgroup Analyses According to AML Type

Adverse risk cytogenetics and secondary AML are unfavorable factors affecting patients’ prognoses. To better understand the clinical outcomes of AML with *RUNX1*^mut^, we focused on AML patients who did not have these dismal prognostic factors. Three subgroup analyses were performed: intermediate-risk cytogenetics, *de novo* AML, and *de novo* AML with intermediate-risk cytogenetics [14,28,29,30,31,33,34,35,36,37,38,41,42,44,46].

For AML patients with intermediate cytogenetic risks, our pooled data showed that patients in the *RUNX1*^wt^ group achieved higher CR rate, with a pooled OR of 0.30 (95% CI: 0.20–0.45; I^2^ = 0%; *p <* 0.00001; Supplementary Data S8) [30,31,33,37]. Even though the OS and EFS rates were similar to those in the primary analysis [30,31,35,37,38,42,45], the RFS of the groups did not significantly differ (pooled RR: 1.25; 95% CI: 0.73–2.12; I^2^ = 70%; *p* = 0.42; Supplementary Data S8) [30,33,37,38,42].

Moving on to the outcomes of the *de novo* AML populations, the CR and OS rates were consistent with the full analysis [14,28,29,33,34,36,37,38,44]. The RFS and EFS rates of the *RUNX1*^mut^ patients did not significantly differ between the groups, with pooled RRs of 1.23 (95% CI: 0.81–1.85; I^2^ = 57%; *p* = 0.33) and 1.25 (95% CI: 0.99–1.58; I^2^ = 0%; *p* = 0.06), respectively (Supplementary Data S9) [14,28,29,33,34,36,37,38,41,44].

**Figure 4:**
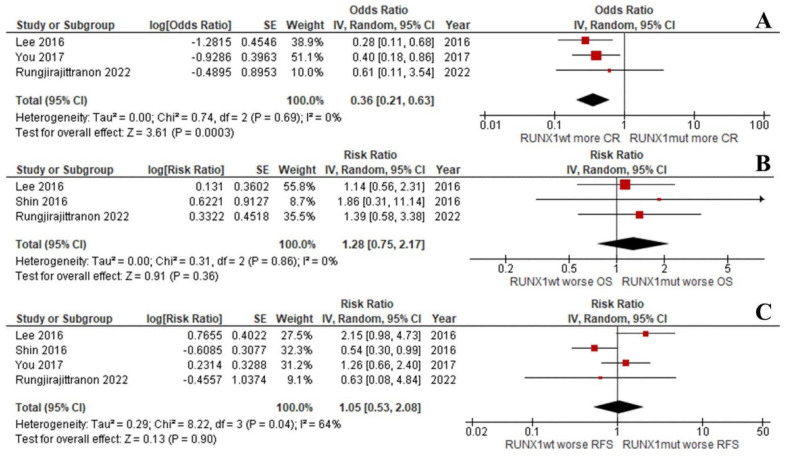
Forest plot of the clinical outcomes of the *RUNX1*^mut^ and *RUNX1*^wt^ *de novo* AML patients with intermediate-risk cytogenetics: (**A**) CR rate; (**B**) OS; (**C**) RFS.

Interestingly, the OS and RFS of the groups were equal when focusing on *de novo* AML patients with intermediate-risk cytogenetics (RR: 1.28; 95% CI: 0.75–2.17; I^2^ = 0%; *p* = 0.36; Figure 4B and RR: 1.05; 95% CI: 0.53–2.08; I^2^ = 64%; *p* = 0.90; Figure 4C) [33,37,38]. This was despite the CR rate being significantly lower for the *RUNX1*^mut^ patients (RR: 0.36; 95% CI: 0.21–0.63; I^2^ = 0%; *p* = 0.0003; Figure 4A) [37,38]. Unfortunately, the number of included studies was insufficient to compare the EFS outcomes of the two groups.

#### 3.2.5. Sensitivity Analysis

A sensitivity analysis was performed by removing studies that had a serious risk of bias according to ROBINS-I. The CR, OS, RFS, and EFS outcomes from non-serious articles were similar to those from the primary analysis (Figure 2 and Figure 3) [9,29,31,33,34,35,37,38,39,40,41,44].

## 4. Discussion

Our prospective study found higher proportions of AML patients with advanced age, secondary AML, and intermediate-risk cytogenetics among those with *RUNX1*^mut^ than among those with *RUNX1*^wt^. These findings are consistent with the results of previous investigations [9,13,15,29,33]. Regarding molecular mutations, the *ASLX1* mutation was found to be a significant co-mutation in the *RUNX1*^mut^ arm. Our data show that *FLT3*-ITD and *TP53* mutations correspond to worse prognostic factors for OS and RFS, in parallel with the risk stratification from the 2017 ELN recommendations [21]. Currently, the *FLT3*-ITD mutation is deemed an intermediate risk by the 2022 ELN recommendations [5]. However, our investigation found that this mutation was associated with poor outcomes. Unfortunately, the patients in our study with the *FLT3*-ITD mutation could not access an FLT3 inhibitor for their treatment because they could not afford the medication.

Furthermore, among the few patients with allogeneic stem cell transplantations in the present investigation’s cohort, the OS and RFS of the patients with *RUNX1*^mut^ were comparable to those with *RUNX1*^wt^. We hypothesize that our results might represent the sole impact of *RUNX1*^mut^ because less than 20% of the AML patients in our cohort had secondary AML, which was an independent risk factor for lower response rates, OS, and EFS [49]. Unlike AML with mutated *TP53,* which is a novel subtype with a homogeneously dismal prognosis, AML patients with *RUNX1*^mut^ seemed to have a heterogeneous prognosis, depending on the host and disease factors [50,51,52,53,54,55].

A systematic review and meta-analysis conducted in 2018 revealed that OS and disease-free survival were almost twice as poor in patients with *RUNX1*^mut^; however, only a few studies were analyzed [12]. In addition, the meta-analysis did not conduct a subgroup analysis of AML patients without poor risk factors, such as adverse cytogenetic risk and secondary AML. Since that meta-analysis, several studies have been published. Our systematic review and meta-analysis gathered all existing studies; 4 to 245 *RUNX1*^mut^ AML patients were in each study cohort, with a median of 26 cases per cohort. The number of *RUNX1*^mut^ AML patients in the present investigation was as high as the median (26) of the included studies. Most of the included studies were conducted on Western populations. To better understand the prognosis of AML with *RUNX1*^mut^ in non-high-risk groups, we conducted the present meta-analysis using the included studies combined with our cohort study’s results to better determine real-world outcomes.

As expected, *RUNX1*^mut^ is a contributing factor to unpleasant prognoses. The *de novo RUNX1*^mut^ AML patients with intermediate-risk cytogenetics had comparable OS and RFS to the *RUNX1*^wt^ group, despite the *RUNX1*^mut^ AML patients having a lower CR rate. As stated in the 2017 and 2022 ELN AML risk stratifications, patients with *RUNX1*^mut^ should undergo allergenic stem cell transplantation [5,8]. Integrated, measurable residual disease (MRD) monitoring was recommended by the European LeukemiaNet MRD Working Party of 2021 as a surrogate marker for guiding the treatment of AML patients [56]. Patients with intermediate-risk cytogenetics will be considered for consolidation chemotherapy or autologous stem cell transplant if their MRD status is negative after two cycles of chemotherapy [56]. Venditti et al. demonstrated outcomes that supported the use of autologous stem cell transplantation in patients with favorable risk and intermediate risk with MRD negativity [57]. Concerning our study outcomes, autologous stem cell transplantation might be the treatment paradigm for *RUNX1*^mut^ *de novo* AML patients with intermediate-risk cytogenetics who achieve negative MRD. This approach would be beneficial in countries where the availability of allogeneic stem cell transplantations is limited.

Our study had some limitations. First, the impact of co-genetic mutations could not be explored either in the prospective study or the meta-analysis. A previous study showed the significant impact of co-mutations on prognosis [4]. Unfortunately, several studies in our meta-analysis did not detail mutations other than *RUNX1*^mut^ nor the impact of co-mutations. Second, some baseline characteristics, such as induction treatment regimens, MRD monitoring, and hematopoietic stem cell transplantation, were missing from observational studies. Third, limited studies were available for our subgroup analyses. Fourth, the subgroup analysis of the outcomes of patients who received either targeted therapy or allogeneic stem cell transplantation could not be evaluated in our meta-analysis due to a lack of data. Additionally, our prospective study included a small number of patients with relatively short follow-up periods. Lastly, we can see from Figure 1 that the hazards in patients with *RUNX1*^wt^ and *RUNX1*^mut^ were not proportionate. This finding opposes our assumption and may have led to an overestimated HR size.

## 5. Conclusions

Owing to the high proportion of secondary AML and elderly patients among AML patients with *RUNX1*^mut^, our study affirmed the poor prognosis of this mutation. However, the survival outcomes of *de novo* AML patients with intermediate-risk cytogenetics in the *RUNX1*^wt^ and *RUNX1*^mut^ groups might be similar.

## Figures and Tables

**Table 1:** Baseline characteristics of patients.

Factor	Total (*N* = 135)	*RUNX1*^mut^ (*N* = 27) (20%)	*RUNX1*^wt^ (*N* = 108) (80%)	*p* Value
**Sex**				0.832
-Female	67 (49.6%)	14 (51.9%)	53 (49.1%)	
-Male	68 (50.4%)	13 (48.1%)	55 (50.9%)	
**Median age (IQR) (years)**	55 (40–64)	62 (55–75)	53 (40–61)	**0.008**
**Comorbidities**				
-Hypertension	37 (34.3%)	7 (29.2%)	30 (35.7%)	0.631
-Diabetes mellitus	20 (18.4%)	4 (16.7%)	16 (18.8%)	1.000
-Chronic kidney disease	2 (1.9%)	0 (0%)	2 (2.4%)	1.000
**ECOG**				0.067
-ECOG 0	4 (3.3%)	1 (5.0%)	3 (3.0%)	
-ECOG 1	100 (83.3%)	13 (65.0%)	87 (87.0%)	
-ECOG 2	12 (10.0%)	5 (25.0%)	7 (7.0%)	
-ECOG 3	3 (2.5%)	1 (5.0%)	2 (2.0%)	
-ECOG 4	1 (0.8%)	0 (0%)	1 (1.0%)	
**Cytogenetic risk**				0.237
-Favorable	11 (8.2%)	0 (0%)	11 (10.2%)	
-Intermediate	99 (73.3%)	22 (81.5%)	77 (71.3%)	
-Adverse	25 (18.5%)	5 (18.5%)	20 (18.5%)	
**Type of AML**				**0.002**
*De novo* AML	110 (81.5%)	16 (59.3%)	94 (87.0%)	
Secondary AML	25 (18.5%)	11 (40.7%)	14 (13.0%)	
**Laboratory at diagnosis**				
**Mean ± SD**				
-Hemoglobin (g/dL)	7.6 ± 2.5	7.7 ± 2.2	7.6 ± 2.6	0.903
**Median ± IQR**				
-WBC (/cumm.)	16,740	5260	23,000	0.731
	(4400–78,070)	(2500–27,890)	(7300–81,110)	
-Platelet (/cumm.)	48,000	38,500	50,000	0.570
	(22,000–115,000)	(14,000–71,000)	(25,000–124,000)	
-Bone marrow blast (%)	69.5	50.0	60.0	0.118
	(34.0–90.0)	(26.0–80.0)	(34.0–90.0)	
**Common molecular mutation (%)**				
-*FLT3*-ITD	30 (22.2%)	6 (22.2%)	24 (22.2%)	1.000
*-NPM1*	22 (17.3%)	0 (0%)	22 (21.8%)	**0.007**
*-TP53*	13 (9.6%)	2 (7.4%)	11 (10.2%)	0.740
-Biallelic *CEBPA*	7 (5.2%)	0 (0%)	7 (6.5%)	0.344
-*DNMT3A*	30 (22.2%)	8 (29.6%)	22 (20.4%)	0.438
*-EZH2*	7 (5.2%)	3 (11.1%)	4 (3.7%)	0.143
*-ASXL1*	6 (4.4%)	4 (14.8%)	2 (1.9%)	**0.015**
*-IDH1*	6 (4.4%)	2 (7.4%)	4 (3.7%)	0.599
*-IDH2*	11 (8.1%)	2 (7.4%)	9 (8.3%)	1.000
**Treatment (%)**				0.083
-Low-intensity treatment	37 (28.7%)	11 (44.0%)	26 (25.0%)	
-High-intensity treatment	92 (71.3%)	15 (56.0%)	78 (75.0%)	
**Allogeneic stem cell transplantation**	16 (11.9%)	2 (7.4%)	14 (13.0%)	0.075
**Treatment response**				
-Complete remission	76/92 (82.6%)	13/19 (68.4%)	63/73 (86.3%)	0.075
-Relapse	52 (38.5%)	14 (51.9%)	38 (35.2%)	0.115

Abbreviations: AML—acute myeloid leukemia; ECOG—Eastern Cooperative Oncology Group; *IQR*—interquartile range; *RUNX1*^mut^—*RUNX1* mutation; *RUNX1*^wt^—*RUNX1* wild type; SD—standard deviation; WBC—white blood cell.

**Table 2:** Factors associated with overall survival and relapse-free survival.

Variables	Overall Survival			Relapse-Free Survival		
	Univariate Analysis					
	HR	95% CI	*p* Value	HR	95% CI	*p* Value
Age group	2.77	1.74–4.41	**<0.001**	2.66	1.22–5.81	**0.014**
Cytogenetic risk	2.43	1.44–4.10	**<0.001**	3.34	1.39–8.01	**0.007**
*RUNX1* mutation	1.42	0.77–2.62	0.268	1.37	0.41–4.62	0.604
*FLT3*-ITD mutation	2.04	1.25–3.34	0.005	3.29	1.50–7.21	**0.003**
*NPM1* mutation	0.97	0.52–1.81	0.927	1.27	0.57–1.27	0.549
Biallelic *CEBPA* mutation	0.48	0.22–1.04	0.062	0.90	0.27–2.98	0.872
*DNMT3A* mutation	1.84	1.08–3.13	**0.024**	1.56	0.54–4.55	0.407
*EZH2* mutation	2.86	1.13–7.21	**0.027**	0.04	0–3394.50	0.794
*IDH1* mutation	3.34	1.33–8.40	**0.010**	1.81	0.24–13.37	0.560
*ASXL1* mutation	1.04	0.33–3.31	0.946	1.08	0.14–7.98	0.935
*SRSF2* mutation	2.01	0.73–5.55	0.180	10.04	2.13–47.18	**0.003**
*TP53* mutation	3.10	1.56–6.17	**<0.001**	1.91	0.45–8.15	0.379
Treatment regimen	2.87	1.76–4.69	**<0.001**	1.54	0.46–5.12	0.474
**Variables**	**Multivariate Analysis**					
	**HR**	**95% CI**	***p* Value**	**HR**	**95% CI**	***p* Value**
Age group	2.80	1.44–5.45	**0.002**	3.11	1.32–7.34	**0.009**
Cytogenetic risk	1.64	0.85–3.17	0.138	2.77	1.10–6.93	0.029 *
*FLT3*-ITD mutation	2.74	1.58–4.74	**<0.001**	4.34	1.89–9.96	**0.001**
Biallelic *CEBPA* mutation	0.43	0.10–1.80	0.248	-	-	-
*DNMT3A* mutation	2.19	1.23–3.89	**0.007**	-	-	-
*EZH2* mutation	3.93	1.46–10.54	**0.007**	-	-	-
*IDH1* mutation	3.72	1.36–10.12	**0.010**	-	-	-
*SRSF2* mutation	2.10	0.69–6.39	0.190	23.06	4.41–120.49	**<0.001**
*TP53* mutation	3.15	1.30–7.59	**0.011**	-	-	-
Treatment regimen	1.35	0.67–2.71	0.400	-	-	-

Remarks: Multivariate analyses were performed using adjusted variable factors with a *p* value < 0.2 by univariate analyses. * Not significant after correcting for multiple hypothesis testing using Bonferroni correction (*p* > 0.016). Abbreviations: CI—confidence interval; ECOG—Eastern Cooperative Oncology Group; HR—Hazard Ratio.

**Table 3:** Patients’ baseline characteristics for studies included in the meta-analysis.

First Author’s Name, Year of Publication [Reference]	Group	No.	Sex (M/F)	Median Age (Years, Range)	Type of AML	Cytogenetic Risk	Molecular Mutations	Induction Treatment	HSCT	Study Period	Type
Tang, 2009 [29]	*RUNX1* ^mut^	62	49/13	62 (15–89)	*De novo* AML	Intermediate: 48 Poor: 8 Unknown: 6	*NPM1*: 3 *CEBPA*: 2 *FLT3*-ITD: 14 *FLT3*-TKD: 6 *NRAS*: 5 *KRAS*: 2 *PTPN11*: 4 *WT1*: 2 *MLL*-PTD: 9	3 + 7 regimen: 330 Low-intensity therapy: 140	11/62	1995–2007	PRO
	*RUNX1* ^wt^	408	217/191	48 (15–90)		Favorable: 59 Intermediate: 279 Poor: 58 Unknown: 12	*NPM1*: 103 *CEBPA*: 64 *FLT3*-ITD: 96 *FLT3*-TKD: 27 *NRAS*: 49 *KRAS*: 14 *PTPN11*: 17 *JAK2*: 4 *KIT*: 14 *WT1*: 30 *MLL*-PTD: 19		85/408		
Schnittger, 2011 [28]	*RUNX1* ^mut^	147	79/169	70.5 (18.3–90.1)	*De novo* AML	Intermediate: 125 Poor: 22	*NPM1*: 1 *CEBPA*: 2 *FLT3*-ITD: 24 *NRAS*: 14	NA	59/449	2005–2009	PRO
	*RUNX1* ^wt^	302	68/133	67.1 (20.4–88.1)		Intermediate: 263 Poor: 39	*NPM1*: 57 *CEBPA*: 39 *FLT3*-ITD: 49 *NRAS*: 46	NA			
Greif, 2012 [31]	*RUNX1* ^mut^	10	9/1	73 (54–78)	*De novo* AML: 8 Unknown: 2	Intermediate (Chromosome- negative AML)	*Monoalleic CEBPA*: 2 *FLT3*-ITD: 3 *FLT3*-TKD: 2 *MLL*-PTD: 3 *NRAS*: 1 *IDH1* or *IDH2*: 2	Intensive Ara-C based regimen	NA	1999–2012	PRO
	*RUNX1* ^wt^	63	26/37	54 (27–83)	*De novo* AML: 57 Unknown: 6		*NPM1*: 42 *Monoalleic CEBPA*: 3 *Bialleic CEBPA*: 6 *FLT3*-ITD: 31 *FLT3*-TKD: 5 *MLL*-PTD: 2 *NRAS*: 9 *KIT*: 1 *IDH1* or *IDH2*: 16				
Grossman, 2012 [32]	*RUNX1* ^mut^	162	NA	NA	NA	Intermediate: 137 Poor: 25	*Monoalleic CEBPA*: 31 *Bialleic CEBPA*: 44 *TP53*: 115 *NPM1*: 282 *FLT3*-ITD: 159 *MLL*-PTD: 57 *ASXL1*: 144	AML-specific intensive treatment regimen (Standard-dose or high-dose cytarabine and anthracycline)	NA	2005–2011	PRO
	*RUNX1* ^wt^	745	NA	NA		NA					
Mendler, 2012 (Same population as Metzeler, 2016, but included only intermediate risk cytogenetics) [30]	*RUNX1* ^mut^	49	28/21	68 (30–81)	NA	Intermediate (Chromosome- negative AML)	*NPM1*: 3 *Monoalleic CEBPA*: 2 *Bialleic CEBPA*: 1 *FLT3*-ITD: 17 *FLT3*-TKD: 1 *WT1*: 5 *DNMT3A*: 12 *ASXL1*: 17 *MLL*-PTD: 3 *IDH1* or *IDH2*: 11 *TET2*: 11	Intensive Ara-C or anthracycline based regimen	NA	NA	PRO
	*RUNX1* ^wt^	343	169/174	61 (18–83)			*NPM1*: 238 *Monoalleic CEBPA*: 26 *Bialleic CEBPA*: 34 *FLT3*-ITD: 125 *FLT3*-TKD: 29 *WT1*: 34 *DNMT3A*: 121 *ASXL1*: 21 *MLL*-PTD: 19 *IDH1* or *IDH2*: 104 *TET2*: 79				
Gaidzik, 2016 [9]	*RUNX1* ^mut^	245	147/98	59.2 (19.2–79.1)	*De novo* AML: 194 Secondary AML: 38 Therapy-related AML: 12	Favorable: 2 Intermediate: 112 Poor: 85 Missing: 46	*NPM1*: 13 *Monoalleic CEBPA*: 10 *Bialleic CEBPA*: 2 *FLT3*-ITD: 47 *FLT3*-TKD: 9 *DNMT3A*: 43 *ASXL1*: 50 *KMT2A*-PTD: 33 *IDH1* or *IDH2*: 59 *BCOR*: 13 *EZH2*: 7 *SRSF2*: 29 *PHF6*: 9 *SF3B1*: 6	3 + 7 regimen or ICE (idarubicin, Ara-C, etoposide) ± ATRA or idarubicin/Ara-C/ATRA ± valproic acid	36/245	1998–2013	PRO
	*RUNX1* ^wt^	2194	1137/1057	53.6 (16.3–84.5)	*De novo* AML: 1920 Secondary AML: 119 Therapy-related AML: 131	Favorable: 303 Intermediate: 463 Poor: 813 Missing: 615	*NPM1*: 651 *Monoalleic CEBPA*: 70 *Bialleic CEBPA*: 86 *FLT3*-ITD: 446 *FLT3*-TKD: 153 *DNMT3A*: 49 *ASXL1*: 129 *KMT2A*-PTD: 68 *IDH1* or *IDH2*: 379 *BCOR*: 19 *EZH2*: 34 *SRSF2*: 50 *PHF6*: 33 *SF3B1*: 21		NA		
Lee, 2016 [37]	*RUNX1* ^mut^	22	NA	53 (15–84)	*De novo* AML	Intermediate	*NPM1*: 142 *CEBPA*: 47 *FLT3*-ITD: 51 *IDH2*: 63 *DNMT3A*: 127 *NRAS*: 85	3 + 7 regimen: 406 Others: 13	NA	NA	RET
	*RUNX1* ^wt^	397	NA								
Shin, 2016 [38]	*RUNX1* ^mut^	5	NA	49.6	*De novo* AML	Intermediate (Chromosome- negative AML)	NA	Intensive chemotherapy: 46 Low-intensity chemotherapy: 6 Supportive treatment: 4	NA	2008–2012	RET
	*RUNX1* ^wt^	51	NA								
Metzeler, 2016 [39]	*RUNX1* ^mut^	102	334/330	57 (18–86)	*De novo* AML: 570 Secondary AML: 59 Therapy-related AML: 35	Favorable: 65 Intermediate: 452 Poor: 129	*NPM1*: 221 Monoalleic *CEBPA*: 25 Bialleic *CEBPA*: 27 *FLT3*-ITD: 197 *DNMT3A*: 209 *TP53*: 63	TAD followed by HAM or HAM for 2 cycles:	NA	1999–2012	RET
	*RUNX1* ^wt^	562									
Lin, 2017 [40]	*RUNX1^mut^*	7	67/45	42.6 (11.7–79)	*De novo* AML	Favorable: 22 Intermediate: 69 Poor: 21	*CEBPA:* 7 *DNMT3A:* 14 *IDH1/2:* 18 *GATA2:* 7 *NPM1:* 17 *WT1:* 13 *FLT3-ITD:* 24 *ASXL1:* 18 *TET:* 12 *TP53:* 9 *KIT:* 5 *NPM1:* 10	3 + 7 regimen: 112	19/112	NA	RET
	*RUNX1* ^wt^	105									
Khan, 2017 [13]	*RUNX1* ^mut^	33	NA	(31–92)	*De novo* AML: 20 Secondary AML: 9 Therapy-related AML: 4	NA	*FLT3*-ITD: 7	Chemotherapy: younger HMAs: elderly	NA	2013–2016	PRO
	*RUNX1* ^wt^	295	NA	(17–91)	*De novo* AML: 246 Secondary AML: 31 Therapy-related AML: 28		*FLT3*-ITD: 55				
Weinberg, 2017 [41]	*RUNX1^mut^*	22	NA	58.5	*De novo* AML	NA	*NPM1:* 56 *CEBPA*: 2	Standard induction chemotherapy: 137	92/137	2009–2015	RET
	*RUNX1* ^wt^	115									
You, 2017 [33]	*RUNX1* ^mut^	33	22/11	61 (12–80)	*De novo* AML	Intermediate	*FLT3*-ITD: 9 *FLT3*-TKD: 1 *MLL*-PTD: 2	3 + 7 regimen	NA	2008–2015	PRO
	*RUNX1* ^wt^	186	102/84	55 (1–84)			*NPM1*: 49 *CEBPA*: 13 *FLT3*-ITD: 51 *FLT3*-TKD: 4 *MLL*-PTD: 11				
Tsai, 2017 [34]	*RUNX1* ^mut^	53	NA	NA	*De novo* AML	Favorable: 55 Intermediate: 263 Poor: 57	*FLT3*-ITD: 86 *FLT3*-TKD: 24 *DNMT3A*: 65 *IDH1: 26* *IDH2: 45* *TET2*: 47 NPM1: 84 *ASXL1: 46* *NRAS: 48* *KRAS: 14* *PTPN11: 18* *KIT: 13* *WT1: 25* *CEBPA: 58* *MLL/PTD: 25* *TP53: 32*	NA	NA	NA	PRO
	*RUNX1* ^wt^	338									
Saygin, 2018 [42]	*RUNX1* ^mut^	22	NA	NA	NA	Intermediate	*FLT3*-ITD: 28 *DNMT3A*: 28 *IDH2:* 18 *TET2*: 18 NPM1: 18 *ASXL1: 16* *U2AF1:16*	3 + 7 regimen	NA	2002–2016	RET
	*RUNX1* ^wt^	126	NA	NA							
Wu, 2018 [35]	*RUNX1* ^mut^	17	57/49	60 (21–88)	NA	Intermediate	*NPM1*: 48 Monoallelic *CEBPA*: 5 Biallelic *CEBPA*: 5 *FLT3*-ITD: 24 *DNMT3A*: 42 *NRAS* or *KRAS*: 14 *IDH1 or IDH2*: 29 *TET2*: 13 *MLL*: 9 *WT1*: 7	NA	44/106	NA	PRO
	*RUNX1* ^wt^	89									
In’t Hout, 2020 [43]	*RUNX1* ^mut^	26	NA	<60 years: 15 ≥60 years: 11	NA	Intermediate: 24 Poor: 2	NA	NA	NA	NA	RET
	*RUNX1* ^wt^	304	NA	<60 years: 249 ≥60 years: 55		Favorable: 87 Intermediate: 165 Poor: 52					
Quesada, 2020 [14]	*RUNX1* ^mut^	46	32/14	66.5 (20–87)	*De novo* AML	Intermediate	*FLT3*-ITD: 19 *DNMT3A*: 10 *ASXL1*: 14 *NRAS*: 13 *IDH2*: 12 *TET2*: 6 *EZH2*: 4 *CEBPA*: 1 *SRSF2*: 9 *SRSF2/SF3B1*: 12	3 + 7 regimen: 20 Nucleoside analogue-based regimen: 10 HMAs: 14 Unknown: 2	15/46	NA	PRO
	*RUNX1* ^wt^	94	45/49	60.5 (20–86)		Favorable: 21 Intermediate: 46 Poor: 27	*FLT3*-ITD: 21 *DNMT3A*: 19 *ASXL1*: 6 *NRAS*: 14 *IDH2*: 18 *TET2*: 10 *EZH2*: 1 *NPM1*: 20 *CEBPA*: 11 *SRSF2*: 1 *SRSF2/SF3B1*: 1	3 + 7 regimen: 34 Nucleoside analogue-based regimen: 30 Adriamycin/daunorubicin/etoposide: 1 Idarubicin/Ara-C/suberoylanilide/hydroxamic acid: 1 HMAs: 24 Unknown: 4	39/94		
Ni, 2020 [36]	*RUNX1* ^mut^	16	52/40	67 (60–75)	*De novo* AML	Favorable: 6 Intermediate: 54 Poor: 15	*FLT3*-ITD: 17 *DNMT3A*: 25 *ASXL1*: 24 *IDH1* or *IDH2*: 30 *NRAS*: 12 *NPM1*: 21 *TET2*: 16 *TP53*: 13	Decitabine combined with G-CSF/Ara-C/idarubicin priming regimen	0	2016–2019	PRO
	*RUNX1* ^wt^	76							0		
Chen, 2021 [44]	*RUNX1* ^mut^	11	103/101	54.5 (20–86)	*De novo* AML	Favorable: 15 Intermediate: 163 Poor: 26	NA	3 + 7 regimen: 135 Homoharringtonine/Ara-C: 49 Low-intensity therapy: 20	NA	NA	RET
	*RUNX1* ^wt^	193									
Ni, 2021 [45]	*RUNX1* ^mut^	17	NA	adults	NA	NA	NA	NA	NA	NA	RET
	*RUNX1* ^wt^	154									
Rehman, 2021 [46]	*RUNX1* ^mut^	8	7/1	65 (52–79)	NA	Intermediate (Chromosome-negative AML)	*FLT3*-ITD: 2 No *FLT3*-ITD: 6	3 + 7 regimen: age <60 years or >60 years with ECOG 0–2 Low-dose Ara-C: >60 years with ECOG 3–4	NA	NA	RET
	*RUNX1* ^wt^	24	19/5	52 (25–82)			*FLT3*-ITD: 1 No *FLT3*-ITD: 23				
Kang, 2022 [47]	*RUNX1* ^mut^	4	29/16	60 (26–87)	Secondary AML: 45	Intermediate: 11 Poor: 34	*FLT3*-ITD: 5 *PTPN11*: 5 *CEBPA:* 5 *SRSF2:* 7 *ASXL1*: 9 *TP53:* 11 *IDH1:* 4 *IDH2:* 9	Intensive chemotherapy: 31 HMA: 5 Low-dose Ara-C: 3	19	2003–2018	RET
	*RUNX1* ^wt^	41									
Current study	*RUNX1* ^mut^	27	13/14	62 (55–75)	*De novo* AML: 16 Secondary AML: 11	Intermediate: 22 Poor: 5	*FLT3*-ITD: 6 *NPM1*: 0 Biallleic *CEBPA*: 0 *ASXL1*: 4	3 + 7 regimen: age < 65 years Low-intensity chemotherapy: age ≥ 65 years	2/27	2018–2021	PRO
	*RUNX1* ^wt^	108	55/53	55 (40–61)	*De novo* AML: 94 Secondary AML: 14	Favorable: 11 Intermediate: 77 Poor: 20	*FLT3*-ITD: 24 *NPM1*: 22 Biallleic *CEBPA*: 7 *ASXL1*: 2		14/108		

Abbreviations: AML—acute myelogenous leukemia; ECOG—Eastern Cooperative Oncology Group; F—female; HMA—hypomethylating agent; HSCT—hematopoietic stem cell transplantation; NA—not available; No.—number; PRO—prospective cohort study; RET—retrospective cohort study; *RUNX1*^mut^—*RUNX1* mutation; *RUNX1*^wt^—*RUNX1* wild type. TAD-9: thioguanine 100 mg/m^2^ twice daily on days 3–9, cytarabine 100 mg/m^2^ continuous infusion on days 1 and 2 and 100 mg/m^2^ twice daily on days 3–8, and daunorubicin 60 mg/m^2^ on days 3–5; HAM: cytarabine 3 g/m^2^ twice daily on days 1–3 and mitoxantrone 10 mg/m^2^ on days 3–5.

## Data Availability

The datasets used and/or analyzed during the current study are available from the corresponding author upon reasonable request.

## Supplementary Materials

The following supporting information can be downloaded at https://www.mdpi.com/article/10.3390/cancers14215239/s1. Supplementary Data S1: Sample size calculation; Supplementary Data S2: Search Strategy; Supplementary Data S3: PRISMA diagram for the systematic review; Supplementary Data S4: Genetic profiling in this cohort study; Supplementary Data S5: The literature review and article selection process; Supplementary Data S6: Funnel plot for the meta-analysis of the risk ratio in OS; Supplementary Data S7: Study quality and risk of bias tool; Supplementary Data S8: Forest plot of the clinical outcomes of the *RUNX1*^mut^ and *RUNX1*^wt^ AML patients with intermediate-risk cytogenetics: (A) CR rate; (B) OS; (C) RFS; (D) EFS; Supplementary Data S9: Forest plot of the clinical outcomes of the *RUNX1*^mut^ and *RUNX1*^wt^ *de novo* AML patients: (A) CR rate; (B) OS; (C) RFS; (D) EFS.

## Abbreviations

AML	acute myeloid leukemia
CR	complete remission
CI	confidence interval
ECOG	Eastern Cooperative Oncology Group
EFS	event-free survival
ELN	European LeukemiaNet
HMAs	hypomethylating agents
HR	hazard ratio
IQR	interquartile range
OR	odds ratio
OS	overall survival
RFS	relapse-free survival
RR	risk ratio
*RUNX1* ^mut^	RUNX1 mutation
*RUNX1* ^wt^	RUNX1 wild type
WHO	World Health Organization

## References

[1] DiNardo C.D., Cortes J.E. (2016). Mutations in AML: Prognostic and therapeutic implications. Hematol. Am. Soc. Hematol. Educ. Program..

[2] Kumar C.C. (2011). Genetic abnormalities and challenges in the treatment of acute myeloid leukemia. Genes Cancer.

[3] Swaminathan M., Morita K., Yuanqing Y., Wang W., Burks J.K., Gumbs C., Little L., Tippen S., Thornton R., Coyle M. (2018). Clinical heterogeneity of acute myeloid leukemia is associated with mutational heterogeneity. Blood.

[4] Papaemmanuil E., Gerstung M., Bullinger L., Gaidzik V.I., Paschka P., Roberts N.D., Potter N.E., Heuser M., Thol F., Bolli N. (2016). Genomic Classification and Prognosis in Acute Myeloid Leukemia. N. Engl. J. Med..

[5] Döhner H., Wei A.H., Appelbaum F.R., Craddock C., DiNardo C.D., Dombret H., Ebert B.L., Fenaux P., Godley L.A., Hasserjian R.P. (2022). Diagnosis and Management of AML in Adults: 2022 ELN Recommendations from an International Expert Panel. Blood.

[6] Okuda T., van Deursen J., Hiebert S.W., Grosveld G., Downing J.R. (1996). AML1, the target of multiple chromosomal translocations in human leukemia, is essential for normal fetal liver hematopoiesis. Cell.

[7] Yokota A., Huo L., Lan F., Wu J., Huang G. (2020). The Clinical, Molecular, and Mechanistic Basis of RUNX1 Mutations Identified in Hematological Malignancies. Mol. Cells.

[8] Arber D.A., Orazi A., Hasserjian R., Thiele J., Borowitz M.J., Le Beau M.M., Bloomfield C.D., Cazzola M., Vardiman J.W. (2016). The 2016 revision to the World Health Organization classification of myeloid neoplasms and acute leukemia. Blood.

[9] Gaidzik V.I., Teleanu V., Papaemmanuil E., Weber D., Paschka P., Hahn J., Wallrabenstein T., Kolbinger B., Köhne C.H., Horst H.A. (2016). RUNX1 mutations in acute myeloid leukemia are associated with distinct clinico-pathologic and genetic features. Leukemia.

[10] Haferlach T., Stengel A., Eckstein S., Perglerová K., Alpermann T., Kern W., Haferlach C., Meggendorfer M. (2016). The new provisional WHO entity ’RUNX1 mutated AML’ shows specific genetics but no prognostic influence of dysplasia. Leukemia.

[11] Khoury J.D., Solary E., Abla O., Akkari Y., Alaggio R., Apperley J.F., Bejar R., Berti E., Busque L., Chan J.K.C. (2022). The 5th edition of the World Health Organization Classification of Haematolymphoid Tumours: Myeloid and Histiocytic/Dendritic Neoplasms. Leukemia.

[12] Jalili M., Yaghmaie M., Ahmadvand M., Alimoghaddam K., Mousavi S.A., Vaezi M., Ghavamzadeh A. (2018). Prognostic Value of RUNX1 Mutations in AML: A Meta-Analysis. Asian Pac. J. Cancer Prev..

[13] Khan M., Cortes J., Kadia T., Naqvi K., Brandt M., Pierce S., Patel K.P., Borthakur G., Ravandi F., Konopleva M. (2017). Clinical Outcomes and Co-Occurring Mutations in Patients with RUNX1-Mutated Acute Myeloid Leukemia. Int. J. Mol. Sci..

[14] Quesada A.E., Montalban-Bravo G., Luthra R., Patel K.P., Sasaki K., Bueso-Ramos C.E., Khoury J.D., Routbort M.J., Bassett R., Hidalgo-Lopez J.E. (2020). Clinico-pathologic characteristics and outcomes of the World Health Organization (WHO) provisional entity de novo acute myeloid leukemia with mutated RUNX1. Mod. Pathol..

[15] Venugopal S., Loghavi S., Dinardo C., Konopleva M., Kadia T., Bhalla K., Issa G.C., Ravandi F., Daver N., Borthakur G. (2021). RUNX1 Mutations in newly diagnosed acute myeloid leukemia do not adversely impact clinical outcomes in the modern era. HemaSphere.

[16] Kent W.J., Sugnet C.W., Furey T.S., Roskin K.M., Pringle T.H., Zahler A.M., Haussler D. (2002). The human genome browser at UCSC. Genome Res..

[17] Sherry S.T., Ward M.H., Kholodov M., Baker J., Phan L., Smigielski E.M., Sirotkin K. (2001). dbSNP: The NCBI database of genetic variation. Nucleic Acids Res..

[18] Tate J.G., Bamford S., Jubb H.C., Sondka Z., Beare D.M., Bindal N., Boutselakis H., Cole C.G., Creatore C., Dawson E. (2019). COSMIC: The Catalogue Of Somatic Mutations In Cancer. Nucleic Acids Res..

[19] Stone R.M., Mandrekar S.J., Sanford B.L., Laumann K., Geyer S., Bloomfield C.D., Thiede C., Prior T.W., Döhner K., Marcucci G. (2017). Midostaurin plus Chemotherapy for Acute Myeloid Leukemia with a FLT3 Mutation. N. Engl. J. Med..

[20] Page M.J., McKenzie J.E., Bossuyt P.M., Boutron I., Hoffmann T.C., Mulrow C.D., Shamseer L., Tetzlaff J.M., Akl E.A., Brennan S.E. (2021). The PRISMA 2020 statement: An updated guideline for reporting systematic reviews. BMJ.

[21] Döhner H., Estey E., Grimwade D., Amadori S., Appelbaum F.R., Büchner T., Dombret H., Ebert B.L., Fenaux P., Larson R.A. (2017). Diagnosis and management of AML in adults: 2017 ELN recommendations from an international expert panel. Blood.

[22] Sterne J.A., Hernán M.A., Reeves B.C., Savović J., Berkman N.D., Viswanathan M., Henry D., Altman D.G., Ansari M.T., Boutron I. (2016). ROBINS-I: A tool for assessing risk of bias in non-randomised studies of interventions. BMJ.

[23] Mathes T., Kuss O. (2018). A comparison of methods for meta-analysis of a small number of studies with binary outcomes. Res. Synth. Methods.

[24] Higgins J.P.T., Thompson S.G., Deeks J.J., Altman D.G. (2003). Measuring inconsistency in meta-analyses. BMJ.

[25] Suurmond R., van Rhee H., Hak T. (2017). Introduction, comparison, and validation of Meta-Essentials: A free and simple tool for meta-analysis. Res. Synth. Methods.

[26] Winer E.S. (2020). Secondary Acute Myeloid Leukemia: A Primary Challenge of Diagnosis and Treatment. Hematol. Oncol. Clin. North Am..

[27] Cheson B.D., Bennett J.M., Kopecky K.J., Büchner T., Willman C.L., Estey E.H., Schiffer C.A., Doehner H., Tallman M.S., Lister T.A. (2003). Revised recommendations of the International Working Group for Diagnosis, Standardization of Response Criteria, Treatment Outcomes, and Reporting Standards for Therapeutic Trials in Acute Myeloid Leukemia. J. Clin. Oncol..

[28] Schnittger S., Dicker F., Kern W., Wendland N., Sundermann J., Alpermann T., Haferlach C., Haferlach T. (2011). RUNX1 mutations are frequent in de novo AML with noncomplex karyotype and confer an unfavorable prognosis. Blood.

[29] Tang J.-L., Hou H.-A., Chen C.-Y., Liu C.-Y., Chou W.-C., Tseng M.-H., Huang C.-F., Lee F.-Y., Liu M.-C., Yao M. (2009). AML1/RUNX1 mutations in 470 adult patients with de novo acute myeloid leukemia: Prognostic implication and interaction with other gene alterations. Blood.

[30] Mendler J.H., Maharry K., Radmacher M.D., Mrózek K., Becker H., Metzeler K.H., Schwind S., Whitman S.P., Khalife J., Kohlschmidt J. (2012). RUNX1 mutations are associated with poor outcome in younger and older patients with cytogenetically normal acute myeloid leukemia and with distinct gene and MicroRNA expression signatures. J. Clin. Oncol..

[31] Greif P.A., Konstandin N.P., Metzeler K.H., Herold T., Pasalic Z., Ksienzyk B., Dufour A., Schneider F., Schneider S., Kakadia P.M. (2012). RUNX1 mutations in cytogenetically normal acute myeloid leukemia are associated with a poor prognosis and up-regulation of lymphoid genes. Haematologica.

[32] Grossmann V., Schnittger S., Kohlmann A., Eder C., Roller A., Dicker F., Schmid C., Wendtner C.-M., Staib P., Serve H. (2012). A novel hierarchical prognostic model of AML solely based on molecular mutations. Blood.

[33] You E., Cho Y.-U., Jang S., Seo E.-J., Lee J.-H., Lee J.-H., Lee K.-H., Koh K.-N., Im H.-J., Seo J.-J. (2017). Frequency and Clinicopathologic Features of RUNX1 Mutations in Patients With Acute Myeloid Leukemia Not Otherwise Specified. Am. J. Clin. Pathol..

[34] Tsai C.-H., Hou H.-A., Tang J.-L., Kuo Y.-Y., Chiu Y.-C., Lin C.-C., Liu C.-Y., Tseng M.-H., Lin T.-Y., Liu M.-C. (2017). Prognostic impacts and dynamic changes of cohesin complex gene mutations in de novo acute myeloid leukemia. Blood Cancer J..

[35] Wu S., Dai Y., Zhang Y., Wang X., Wang L., Ma D., Zhang L., Pang Y., Jiao Y., Niu M. (2018). Mutational spectrum and prognostic stratification of intermediate-risk acute myeloid leukemia. Cancer Gene Ther..

[36] Ni J., Hong J., Long Z., Li Q., Xia R., Zeng Q. (2020). Mutation profile and prognostic relevance in elderly patients with de novo acute myeloid leukemia treated with decitabine-based chemotherapy. Int. J. Lab. Hematol..

[37] Lee S.-S., Ahn J.-S., Kim T., Kim H.-J., Kim Y.-K., Ahn S.-Y., Jung S.-H., Yang D.-H., Lee J.-J., Park H.J. (2016). RUNX1 Mutation in Cytogenetically Normal Acute Myeloid Leukaemia: Clinical Implications, Co-Mutation Analysis. Blood.

[38] Shin S.-Y., Lee S.-T., Kim H.-J., Cho E.H., Kim J.-W., Park S., Jung C.W., Kim S.-H. (2016). Mutation profiling of 19 candidate genes in acute myeloid leukemia suggests significance of DNMT3A mutations. Oncotarget.

[39] Metzeler K.H., Herold T., Rothenberg-Thurley M., Amler S., Sauerland M.C., Görlich D., Schneider S., Konstandin N.P., Dufour A., Bräundl K. (2016). Spectrum and prognostic relevance of driver gene mutations in acute myeloid leukemia. Blood.

[40] Lin P.-H., Li H.-Y., Fan S.-C., Yuan T.-H., Chen M., Hsu Y.-H., Yang Y.-H., Li L.-Y., Yeh S.-P., Bai L.-Y. (2017). A targeted next-generation sequencing in the molecular risk stratification of adult acute myeloid leukemia: Implications for clinical practice. Cancer Med..

[41] Weinberg O.K., Gibson C.J., Blonquist T.M., Neuberg D., Pozdnyakova O., Kuo F., Ebert B.L., Hasserjian R.P. (2017). NPM1 mutation but not RUNX1 mutation or multilineage dysplasia defines a prognostic subgroup within de novo acute myeloid leukemia lacking recurrent cytogenetic abnormalities in the revised 2016 WHO classification. Am. J. Hematol..

[42] Saygin C., Hirsch C., Przychodzen B., Sekeres M.A., Hamilton B.K., Kalaycio M., Carraway H.E., Gerds A.T., Mukherjee S., Nazha A. (2018). Mutations in DNMT3A, U2AF1, and EZH2 identify intermediate-risk acute myeloid leukemia patients with poor outcome after CR1. Blood Cancer J..

[43] Hout F.E.M.I., Gerritsen M., Bullinger L., Van der Reijden B.A., Huls G., Vellenga E., Jansen J.H. (2020). Transcription factor 4 (TCF4) expression predicts clinical outcome in RUNX1 mutated and translocated acute myeloid leukemia. Haematologica.

[44] Chen X., Zhu H., Qiao C., Zhao S., Liu L., Wang Y., Jin H., Qian S., Wu Y. (2021). Next-generation sequencing reveals gene mutations landscape and clonal evolution in patients with acute myeloid leukemia. Hematology.

[45] Ni Z.F., Ma L.J., Shi L.L., Shen P.L., Zhao J.Q. (2021). [Clinical Characteristics of Acute Myeloid Leukemia Patients with RUNX1 Gene Mutation]. Zhongguo Shi Yan Xue Ye Xue Za Zhi..

[46] Rehman A., Akram A.M., Chaudhary A., Sheikh N., Hussain Z., Alsanie W.F., Rehman R.A., Hameed N., Saleem T., Zafar A. (2021). RUNX1 mutation and elevated FLT3 gene expression cooperates to induce inferior prognosis in cytogenetically normal acute myeloid leukemia patients. Saudi J. Biol. Sci..

[47] Kang D., Jung J., Park S., Cho B.-S., Kim H.-J., Kim Y., Lee J.-M., Kim H.S., Ahn A., Kim M. (2022). Genetic Characteristics According to Subgroup of Acute Myeloid Leukemia with Myelodysplasia-Related Changes. J. Clin. Med..

[48] Sterne J.A.C., Sutton A.J., Ioannidis J.P.A., Terrin N., Jones D.R., Lau J., Carpenter J., Rücker G., Harbord R.M., Schmid C.H. (2011). Recommendations for examining and interpreting funnel plot asymmetry in meta-analyses of randomised controlled trials. BMJ.

[49] Cheung E., Perissinotti A.J., Bixby D.L., Burke P.W., Pettit K.M., Benitez L.L., Brown J., Scappaticci G.B., Marini B.L. (2019). The leukemia strikes back: A review of pathogenesis and treatment of secondary AML. Ann. Hematol..

[50] Rücker F.G., Schlenk R.F., Bullinger L., Kayser S., Teleanu V., Kett H., Habdank M., Kugler C.M., Holzmann K., Gaidzik V.I. (2012). TP53 alterations in acute myeloid leukemia with complex karyotype correlate with specific copy number alterations, monosomal karyotype, and dismal outcome. Blood.

[51] Middeke J.M., Herold S., Rücker-Braun E., Berdel W.E., Stelljes M., Kaufmann M., Schäfer-Eckart K., Baldus C.D., Stuhlmann R., Ho A.D. (2016). TP53 mutation in patients with high-risk acute myeloid leukaemia treated with allogeneic haematopoietic stem cell transplantation. Br. J. Haematol..

[52] Montalban-Bravo G., Benton C.B., Wang S.A., Ravandi F., Kadia T., Cortes J., Daver N., Takahashi K., DiNardo C., Jabbour E. (2017). More than 1 TP53 abnormality is a dominant characteristic of pure erythroid leukemia. Blood.

[53] Short N.J., Montalban-Bravo G., Hwang H., Ning J., Franquiz M.J., Kanagal-Shamanna R., Patel K.P., DiNardo C.D., Ravandi F., Garcia-Manero G. (2020). Prognostic and therapeutic impacts of mutant TP53 variant allelic frequency in newly diagnosed acute myeloid leukemia. Blood Adv..

[54] Grob T., Al Hinai A.S.A., Sanders M.A., Kavelaars F.G., Rijken M., Gradowska P.L., Biemond B.J., Breems D.A., Maertens J., van Marwijk Kooy M. (2022). Molecular characterization of mutant TP53 acute myeloid leukemia and high-risk myelodysplastic syndrome. Blood.

[55] Weinberg O.K., Siddon A., Madanat Y.F., Gagan J., Arber D.A., Cin P.D., Narayanan D., Ouseph M.M., Kurzer J.H., Hasserjian R.P. (2022). TP53 mutation defines a unique subgroup within complex karyotype de novo and therapy-related MDS/AML. Blood Adv..

[56] Heuser M., Freeman S.D., Ossenkoppele G.J., Buccisano F., Hourigan C.S., Ngai L.L., Tettero J.M., Bachas C., Baer C., Béné M.-C. (2021). 2021 Update on MRD in acute myeloid leukemia: A consensus document from the European LeukemiaNet MRD Working Party. Blood.

[57] Venditti A., Piciocchi A., Candoni A., Melillo L., Calafiore V., Cairoli R., de Fabritiis P., Storti G., Salutari P., Lanza F. (2019). GIMEMA AML1310 trial of risk-adapted, MRD-directed therapy for young adults with newly diagnosed acute myeloid leukemia. Blood.

