# Supplementary material for: Clinical Outcomes of Acute Myeloid Leukemia Patients Harboring the RUNX1 Mutation: Is It Still an Unfavorable Prognosis? A Cohort Study and Meta-Analysis
Source: Cancers (Basel). 2022 Oct 26;14(21):5239. doi: 10.3390/cancers14215239 (PMC9659296; doi:10.3390/cancers14215239)
Supplement: Supplementary file 1 [file cancers-14-05239-s001.zip › Supplementary data S1_sample size.pdf]

Supplementary Data S1. The sample size calculation.

We calculated sample size based on overall survival (OS) rate from our pilot study in 2017-2019 which was our primary outcome. The study showed that 1-year OS of AML patients with *RUNX1*^{mut} and *RUNX1*^{wt} were 35% and 75%, respectively. Log-rank test was used to compare OS from both groups. So, the number of patients that had to enroll in our study was 27 patients. However, due to 4-fold larger number of patients in unmutated group, we included 108 patients in *RUNX1*^{wt} arm.

Hazard ratio = 3.649 = ln(¶$_1$)/ln(¶$_2$) = ln(0.35)/ln(0.75)

$$n/group = \frac{(Z_{1-\alpha/s} + Z_{1-\beta})^2 (h+1)^2}{(2-\pi_1-\pi_2)(h-1)^2}$$

$$\frac{n}{group} = \frac{(1.96+0.84)^2(3.649+1)^2}{(2-0.35-0.75)(3.649-1)^2}$$

N per group = 27
