# Supplementary material for: Clinical Outcomes of Acute Myeloid Leukemia Patients Harboring the RUNX1 Mutation: Is It Still an Unfavorable Prognosis? A Cohort Study and Meta-Analysis
Source: Cancers (Basel). 2022 Oct 26;14(21):5239. doi: 10.3390/cancers14215239 (PMC9659296; doi:10.3390/cancers14215239)
Supplement: Supplementary file 1 [file cancers-14-05239-s001.zip › Supplementary data S2 Search Strategy (25.09.22).pdf]

Supplementary Data S2. Search Strategy.

MEDLINE

1. "leukemia, myeloid, acute"[MeSH Terms]
2. "leukemia"[All Fields] AND "myeloid"[All Fields] AND "acute"[All Fields])
3. "acute myeloid leukemia"[All Fields]
4. "leukemia"[All Fields] AND "myeloid"[All Fields] AND "acute"[All Fields])
5. "leukemia myeloid acute"[All Fields]
6. "genetic"[All Fields]
7. "molecular"[All Fields]
8. "runx1"[All Fields]
9. "runx1 translocation partner 1 protein"[MeSH Terms]
10. "runx1 translocation partner 1 protein"[All Fields]
11. #1 OR #2 OR #3 OR #4 OR #5
12. #6 OR #7 OR #8 OR #9 OR #10
13. #11 AND #12

Embase

1. 'acute myeloid leukemia'/exp OR 'acute myeloid leukemia'
2. runx1
3. 'molecular genetics'
4. genetic
5. #2 OR #3 OR #4
6. #1 AND #5

Cochrane Library

1. Leukemia, Myeloid, Acute
2. RUNX1 Translocation Partner 1 Protein
3. Genetics

4. #2 OR #3
5. #1 AND #4
