# Supplementary material for: Clinical Outcomes of Acute Myeloid Leukemia Patients Harboring the RUNX1 Mutation: Is It Still an Unfavorable Prognosis? A Cohort Study and Meta-Analysis
Source: Cancers (Basel). 2022 Oct 26;14(21):5239. doi: 10.3390/cancers14215239 (PMC9659296; doi:10.3390/cancers14215239)
Supplement: Supplementary file 1 [file cancers-14-05239-s001.zip › Supplementary data S4_Molecular mutations.pdf]

Supplementary Data S4. Genetic profiling in this cohort study.

**Molecular mutations**	**Total (N=135)**	***RUNX1*^{mut} (N=27) (20%)**	***RUNX1*^{wt} (N=108) (80%)**	***P* value**
-*ASXL1*	6 (4.4%)	4 (14.8%)	2 (1.9%)	**0.015**
-*CBL*	3 (2.2%)	0 (0%)	3 (2.8%)	1.000
-Biallelic *CEBPA*	7 (5.2%)	0 (0%)	7 (6.5%)	0.344
-*CSF3R*	5 (3.7%)	2 (7.4%)	3 (2.8%)	0.264
-*DNMT3A*	30 (22.2%)	8 (29.6%)	22 (20.4%)	0.438
-*EZH2*	7 (5.2%)	3 (11.1%)	4 (3.7%)	0.143
-*FLT3*-ITD	30 (22.2%)	6 (22.2%)	24 (22.2%)	1.000
-*IDH1*	6 (4.4%)	2 (7.4%)	4 (3.7%)	0.599
-*IDH2*	11 (8.1%)	2 (7.4%)	9 (8.3%)	1.000
-*JAK2*	4 (3%)	1 (3.7%)	3 (2.8%)	1.000
-*KIT*	18 (13.3%)	2 (7.4%)	6 (14.8%)	0.526
-*KRAS*	8 (5.9%)	2 (7.4%)	6 (5.6%)	0.660
-*MPL*	5 (3.7%)	0 (0%)	5 (4.6%)	0.583
-*NPM1*	22 (17.3%)	0 (0%)	22 (21.8%)	**0.007**
-*NRAS*	15 (11.2%)	5 (18.5%)	10 (9.3%)	0.183
-*SETBP1*	2 (2.2%)	1 (4.8%)	1 (1.4%)	0.406
-*SF3B1*	3 (3.3%)	3 (14.3%)	0 (0%)	**0.011**
-*SRSF2*	6 (4.5%)	4 (14.8%)	2 (1.9%)	**0.015**
-*TET2*	26 (19.4%)	3 (11.1%)	23 (21.5%)	0.284
-*TP53*	13 (9.6%)	2 (7.4%)	11 (10.2%)	0.740
-*U2AF1*	7 (5.2%)	2 (7.4%)	5 (4.7%)	0.628
-*ZRSR2*	1 (0.8%)	0 (0%)	1 (0.9%)	1.000
