# Supplementary material for: Clinical Outcomes of Acute Myeloid Leukemia Patients Harboring the RUNX1 Mutation: Is It Still an Unfavorable Prognosis? A Cohort Study and Meta-Analysis
Source: Cancers (Basel). 2022 Oct 26;14(21):5239. doi: 10.3390/cancers14215239 (PMC9659296; doi:10.3390/cancers14215239)
Supplement: Supplementary file 1 [file cancers-14-05239-s001.zip › Supplementary data S7_ROB.pdf]

Supplementary Data S7. Study quality and risk of bias tool.

Risk of bias domains

Study	D1	D2	D3	D4	D5	D6	D7	Overal
Tang 2009	-	-	+	+	-	+	-	-
Schnittger 2011	-	-	+	+	-	+	-	-
Greif 2012	-	-	+	+	-	+	-	-
Grossman 2012	X	-	+	+	?	+	X	X
Mendler 2012	-	-	+	+	-	+	-	-
Gaidzik 2016	-	-	+	+	-	+	-	-
Lee 2016	?	-	-	+	?	+	?	?
Metzeler 2016	-	-	+	+	-	+	-	-
Shin 2016	?	-	+	+	-	+	-	-
Khan 2017	X	-	+	+	+	+	-	X
Lin 2017	-	-	+	+	+	+	-	-
Tsai 2017	-	-	+	+	-	+	-	-
Weinberg 2017	-	-	+	+	?	+	-	-
You 2017	-	-	+	+	X	+	-	-
Saygin 2018	X	-	+	+	?	+	-	X
Wu 2018	-	-	+	+	+	+	-	-
In't Hout 2020	X	-	-	+	-	+	-	X
Ni 2020	X	-	+	+	?	+	X	X
Quesada 2020	X	-	+	+	+	+	-	X
Chen 2021	-	-	+	+	+	+	-	-
Ni 2021	X	-	+	+	-	+	X	X
Rehman 2021	X	-	-	+	?	+	X	X
Kang 2022	X	-	+	+	-	+	X	X
Rungjirajittranon 2022	-	-	+	+	+	+	-	-

Domains:
D1: Bias due to confounding.
D2: Bias due to selection of participants.
D3: Bias in classification of interventions.
D4: Bias due to deviations from intended interventions.
D5: Bias due to missing data.
D6: Bias in measurement of outcomes.
D7: Bias in selection of the reported result.

Judgement
X Serious
- Moderate
+ Low
? No information
