# Supplementary material for: Clinical Outcomes of Acute Myeloid Leukemia Patients Harboring the RUNX1 Mutation: Is It Still an Unfavorable Prognosis? A Cohort Study and Meta-Analysis
Source: Cancers (Basel). 2022 Oct 26;14(21):5239. doi: 10.3390/cancers14215239 (PMC9659296; doi:10.3390/cancers14215239)

Supplementary Data S8. Forest plot of the clinical outcomes of the *RUNX1*^{mut} and *RUNX1*^{wt} AML patients with intermediate-risk cytogenetics (A) CR rate; (B) OS; (C) RFS; (D) EFS.

A

Study or Subgroup	log[Odds Ratio]	SE	Weight	Odds Ratio IV, Random, 95% CI	Year
Mendler 2012	-1.3976	0.3279	40.1%	0.25 [0.13, 0.47]	2012
Greif 2012	0.1031	1.1891	3.0%	1.11 [0.11, 11.40]	2012
Lee 2016	-1.2815	0.4546	20.8%	0.28 [0.11, 0.68]	2016
You 2017	-0.9286	0.3963	27.4%	0.40 [0.18, 0.86]	2017
Rungjirajittranon 2022	-1.495	0.7079	8.6%	0.22 [0.06, 0.90]	2022
**Total (95% CI)**			**100.0%**	**0.30 [0.20, 0.45]**	

Heterogeneity: Tau² = 0.00; Chi² = 2.24, df = 4 (P = 0.69); I² = 0%
Test for overall effect: Z = 5.82 (P < 0.00001)

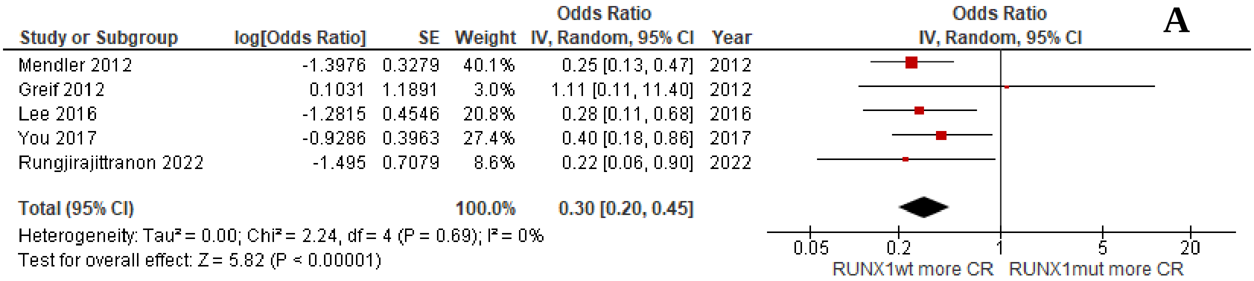

B

Study or Subgroup	log[Risk Ratio]	SE	Weight	Risk Ratio IV, Random, 95% CI	Year
Mendler 2012	0.503	0.1943	33.0%	1.65 [1.13, 2.42]	2012
Greif 2012	0.9266	0.4241	9.4%	2.53 [1.10, 5.80]	2012
Lee 2016	0.131	0.3602	12.6%	1.14 [0.56, 2.31]	2016
Shin 2016	0.6221	0.9127	2.2%	1.86 [0.31, 11.14]	2016
Wu 2018	0.9497	0.3383	14.0%	2.58 [1.33, 5.02]	2018
Saygin 2018	-0.0796	0.4143	9.8%	0.92 [0.41, 2.08]	2018
Rehman 2021	0.0646	0.3095	16.3%	1.07 [0.58, 1.96]	2021
Rungjirajittranon 2022	-0.1495	0.8308	2.6%	0.86 [0.17, 4.39]	2022
**Total (95% CI)**			**100.0%**	**1.51 [1.16, 1.98]**	

Heterogeneity: Tau² = 0.02; Chi² = 8.00, df = 7 (P = 0.33); I² = 12%
Test for overall effect: Z = 3.04 (P = 0.002)

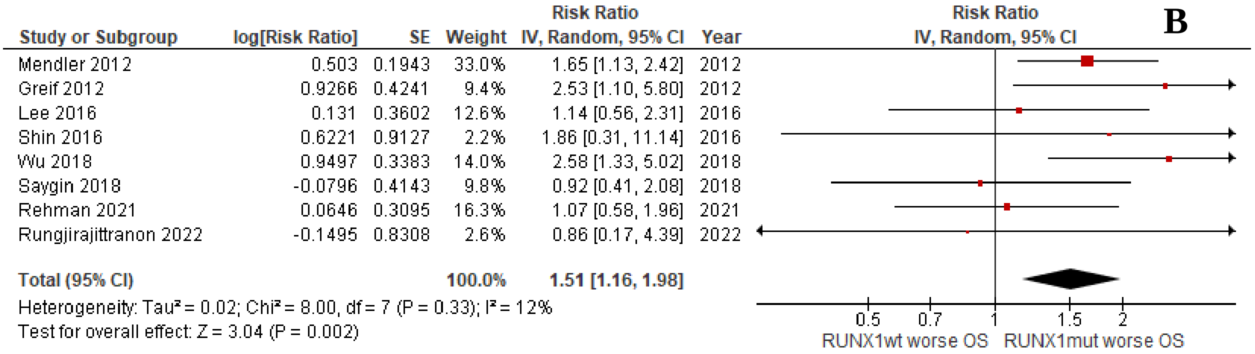

C

Study or Subgroup	log[Risk Ratio]	SE	Weight	Risk Ratio IV, Random, 95% CI	Year
Mendler 2012	0.8481	0.2293	21.8%	2.34 [1.49, 3.66]	2012
Lee 2016	0.7655	0.4022	16.5%	2.15 [0.98, 4.73]	2016
Shin 2016	-0.6085	0.3077	19.4%	0.54 [0.30, 0.99]	2016
You 2017	0.2314	0.3288	18.7%	1.26 [0.66, 2.40]	2017
Saygin 2018	0.0344	0.3512	18.0%	1.03 [0.52, 2.06]	2018
Rungjirajittranon 2022	-0.4262	1.0284	5.5%	0.65 [0.09, 4.90]	2022
**Total (95% CI)**			**100.0%**	**1.25 [0.73, 2.12]**	

Heterogeneity: Tau² = 0.28; Chi² = 16.88, df = 5 (P = 0.005); I² = 70%
Test for overall effect: Z = 0.81 (P = 0.42)

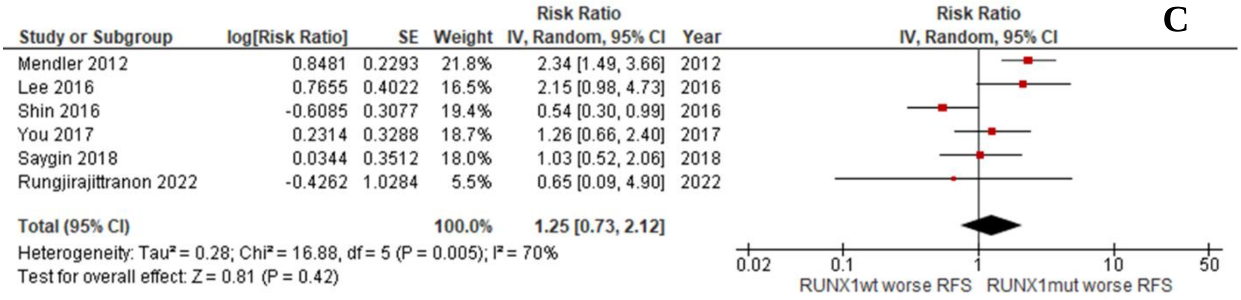

D

Study or Subgroup	log[Risk Ratio]	SE	Weight	Risk Ratio IV, Random, 95% CI	Year
Mendler 2012	0.8193	0.1625	49.6%	2.27 [1.65, 3.12]	2012
Lee 2016	0.392	0.3674	18.7%	1.48 [0.72, 3.04]	2016
Wu 2018	0.6051	0.328	22.2%	1.83 [0.96, 3.48]	2018
Rehman 2021	-0.2513	0.5563	9.4%	0.78 [0.26, 2.31]	2021
**Total (95% CI)**			**100.0%**	**1.81 [1.27, 2.57]**	

Heterogeneity: Tau² = 0.04; Chi² = 4.20, df = 3 (P = 0.24); I² = 29%
Test for overall effect: Z = 3.27 (P = 0.001)

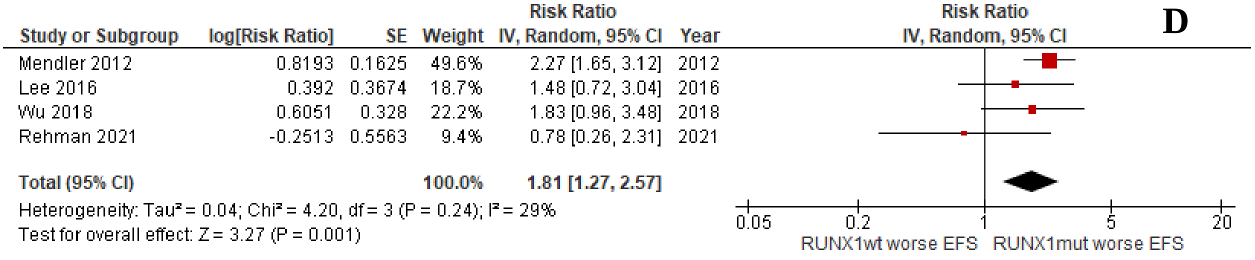

Supplement: Supplementary file 1 [file cancers-14-05239-s001.zip › Supplementary data S8 (11.10.22).pdf]
