# Supplementary material for: Clinical Outcomes of Acute Myeloid Leukemia Patients Harboring the RUNX1 Mutation: Is It Still an Unfavorable Prognosis? A Cohort Study and Meta-Analysis
Source: Cancers (Basel). 2022 Oct 26;14(21):5239. doi: 10.3390/cancers14215239 (PMC9659296; doi:10.3390/cancers14215239)

Supplementary Data S9. Forest plot of the clinical outcomes of the *RUNX1*^{mut} and *RUNX1*^{wt} *de novo* AML patients (A) CR rate; (B) OS; (C) RFS; (D) EFS.

A

Study or Subgroup	log[Odds Ratio]	SE	Weight	Odds Ratio IV, Random, 95% CI	Year
Tang 2009	-0.8956	0.2863	48.6%	0.41 [0.23, 0.72]	2009
Lee 2016	-1.2815	0.4546	19.3%	0.28 [0.11, 0.68]	2016
You 2017	-0.9286	0.3963	25.4%	0.40 [0.18, 0.86]	2017
Rungjirajittranon 2022	-0.8835	0.7666	6.8%	0.41 [0.09, 1.86]	2022
**Total (95% CI)**			**100.0%**	**0.38 [0.25, 0.56]**	

Heterogeneity: Tau² = 0.00; Chi² = 0.56, df = 3 (P = 0.91); I² = 0%
Test for overall effect: Z = 4.90 (P < 0.00001)

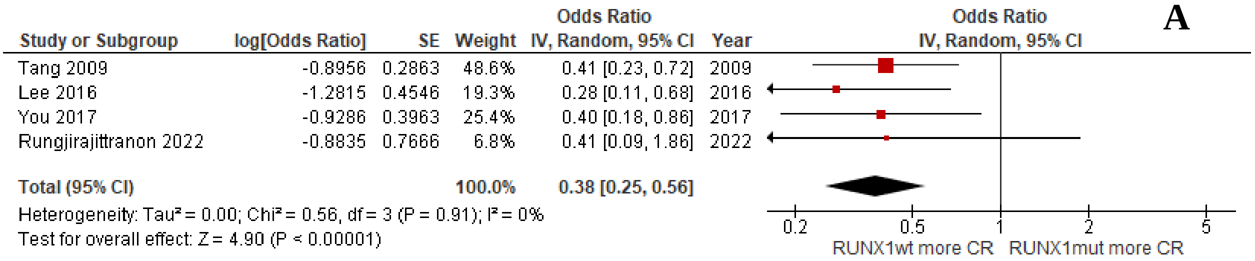

B

Study or Subgroup	log[Risk Ratio]	SE	Weight	Risk Ratio IV, Random, 95% CI	Year
Tang 2009	0.6302	0.2688	15.3%	1.88 [1.11, 3.18]	2009
Schnittger 2011	0.4644	0.2125	24.5%	1.59 [1.05, 2.41]	2011
Shin 2016	0.6221	0.9127	1.3%	1.86 [0.31, 11.14]	2016
Lee 2016	0.131	0.3602	8.5%	1.14 [0.56, 2.31]	2016
Tsai 2017	0.0122	0.2728	14.9%	1.01 [0.59, 1.73]	2017
Ni 2020	0.133	0.4844	4.7%	1.14 [0.44, 2.95]	2020
Quesada 2020	0.0606	0.2383	19.5%	1.06 [0.67, 1.69]	2020
Chen 2021	0.3685	0.4866	4.7%	1.45 [0.56, 3.75]	2021
Rungjirajittranon 2022	0.46	0.4145	6.4%	1.58 [0.70, 3.57]	2022
**Total (95% CI)**			**100.0%**	**1.35 [1.09, 1.65]**	

Heterogeneity: Tau² = 0.00; Chi² = 4.86, df = 8 (P = 0.77); I² = 0%
Test for overall effect: Z = 2.82 (P = 0.005)

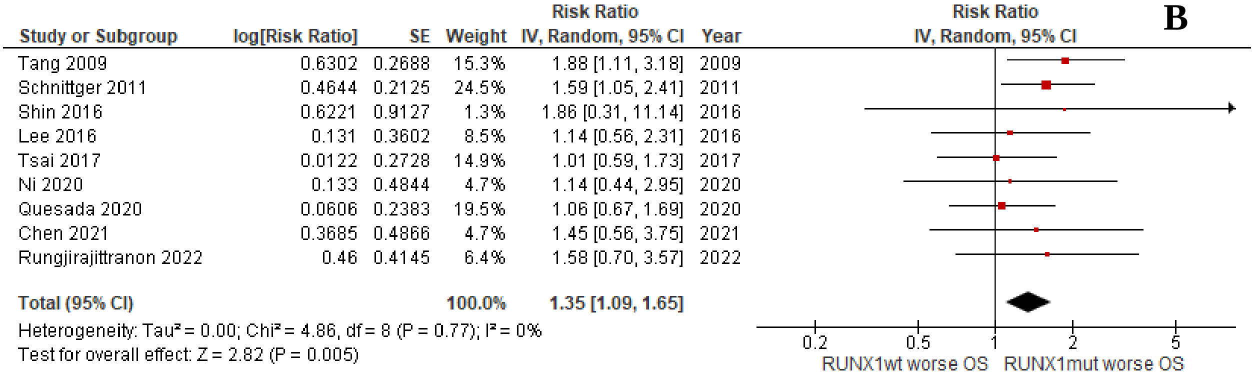

C

Study or Subgroup	log[Risk Ratio]	SE	Weight	Risk Ratio IV, Random, 95% CI	Year
Tang 2009	0.3552	0.2227	21.2%	1.43 [0.92, 2.21]	2009
Lee 2016	0.7655	0.4022	13.8%	2.15 [0.98, 4.73]	2016
Shin 2016	-0.6085	0.3077	17.4%	0.54 [0.30, 0.99]	2016
Tsai 2017	-0.0229	0.255	19.8%	0.98 [0.59, 1.61]	2017
You 2017	0.2314	0.3288	16.5%	1.26 [0.66, 2.40]	2017
Chen 2021	1.4379	0.6449	7.7%	4.21 [1.19, 14.91]	2021
Rungjirajittranon 2022	-0.414	1.0288	3.6%	0.66 [0.09, 4.96]	2022
**Total (95% CI)**			**100.0%**	**1.23 [0.81, 1.85]**	

Heterogeneity: Tau² = 0.16; Chi² = 14.07, df = 6 (P = 0.03); I² = 57%
Test for overall effect: Z = 0.97 (P = 0.33)

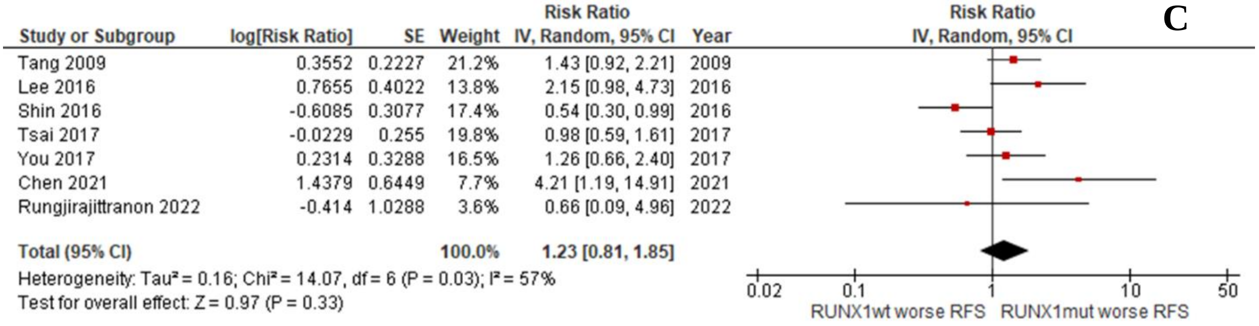

D

Study or Subgroup	log[Risk Ratio]	SE	Weight	Risk Ratio IV, Random, 95% CI	Year
Schnittger 2011	0.2717	0.1924	38.1%	1.31 [0.90, 1.91]	2011
Lee 2016	0.392	0.3674	10.4%	1.48 [0.72, 3.04]	2016
Weinberg 2017	0.2342	0.3238	13.4%	1.26 [0.67, 2.38]	2017
Ni 2020	0.464	0.4668	6.5%	1.59 [0.64, 3.97]	2020
Quesada 2020	0.0513	0.2111	31.6%	1.05 [0.70, 1.59]	2020
**Total (95% CI)**			**100.0%**	**1.25 [0.99, 1.58]**	

Heterogeneity: Tau² = 0.00; Chi² = 1.20, df = 4 (P = 0.88); I² = 0%
Test for overall effect: Z = 1.87 (P = 0.06)

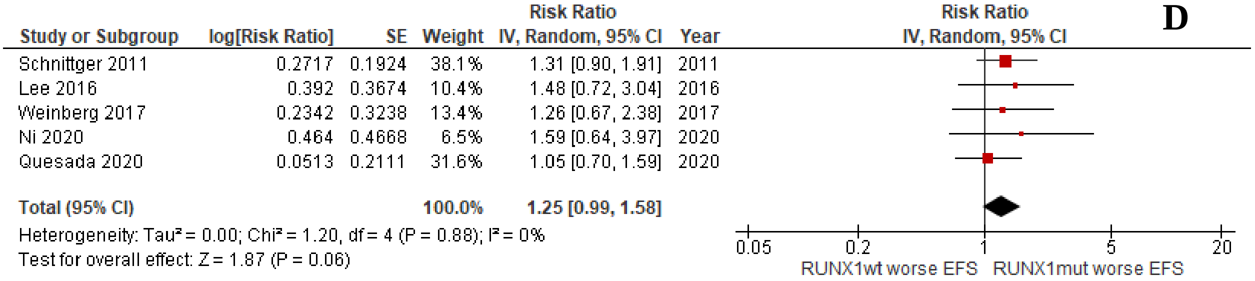

Supplement: Supplementary file 1 [file cancers-14-05239-s001.zip › Supplementary data S9 (11.10.22).pdf]
