# Supplementary figures and images for: Clinical Outcomes of Acute Myeloid Leukemia Patients Harboring the RUNX1 Mutation: Is It Still an Unfavorable Prognosis? A Cohort Study and Meta-Analysis
Source: Cancers (Basel). 2022 Oct 26;14(21):5239. doi: 10.3390/cancers14215239 (PMC9659296; doi:10.3390/cancers14215239)

**Supplementary Data S5.** The literature review and article selection process.

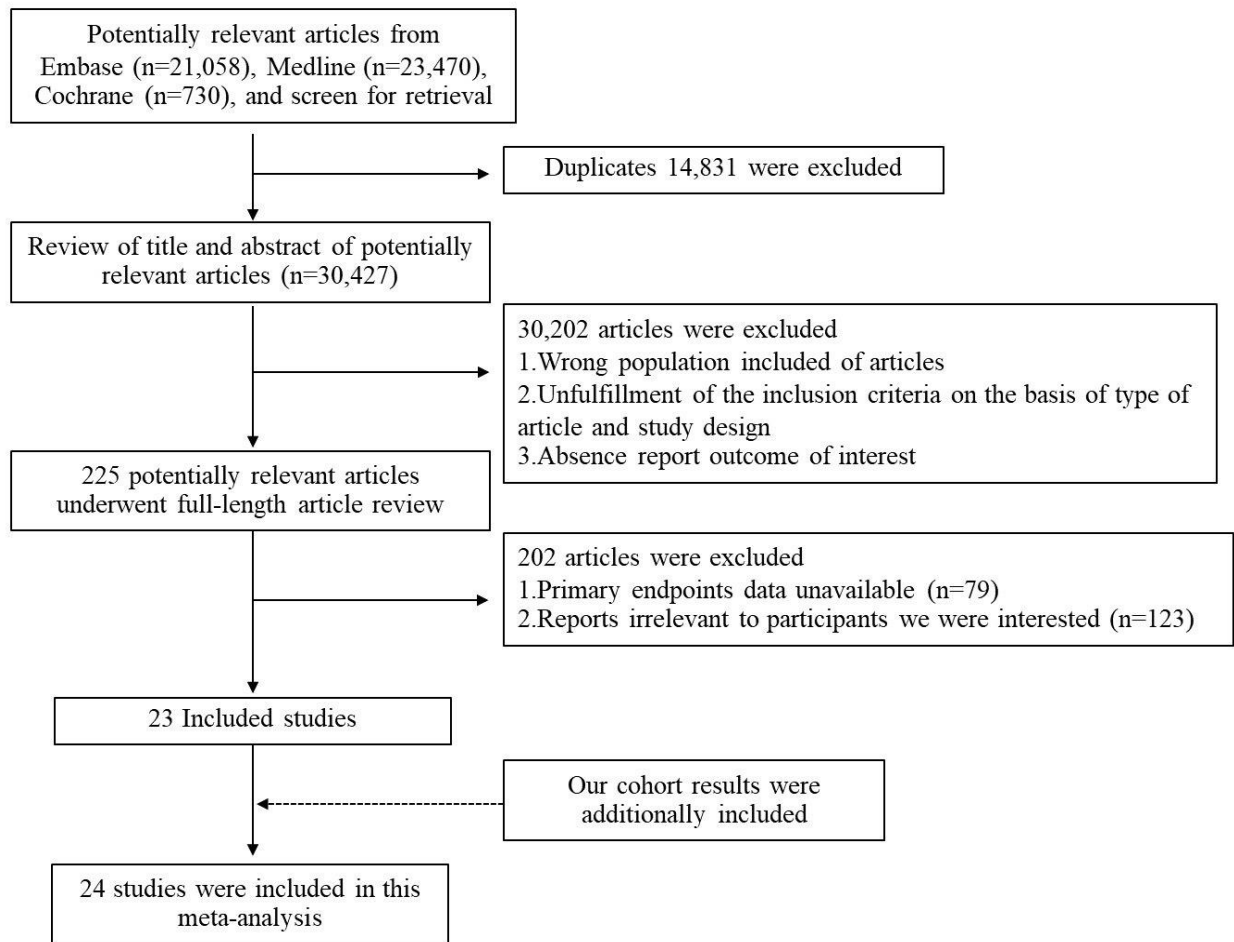

Supplement: Supplementary file 1 [file cancers-14-05239-s001.zip › Supplementary data S5_literaturereview.pdf]

**Supplementary Data S6.** Funnel plot for the meta-analysis of the risk ratio in OS.

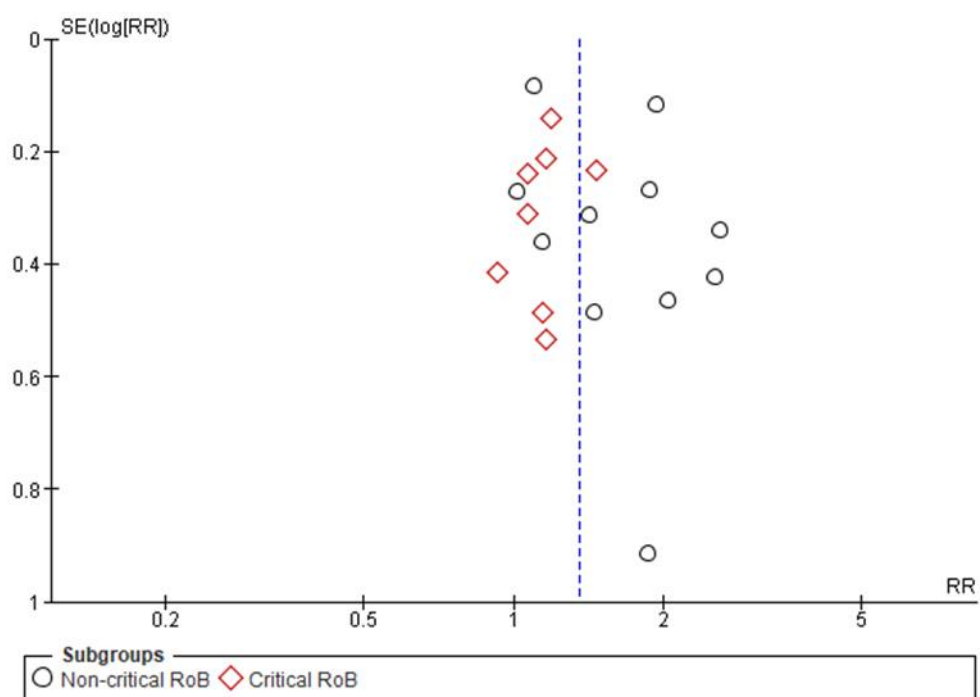

Supplement: Supplementary file 1 [file cancers-14-05239-s001.zip › Supplementary data S6 Funnel plots (25.09.22).pdf]
